# Supplementary material for: Comprehensive assessment of on- and off-target mutagenesis via lipid nanoparticle delivery of CRISPR-Cas9 genome editing
Source: Mol Ther Nucleic Acids. 2026 May 20;37(2):102958. doi: 10.1016/j.omtn.2026.102958 (PMC13267559; doi:10.1016/j.omtn.2026.102958)
Supplement: Document S1. Figures S1–S8 [file mmc1.pdf]

**OMTN, Volume *37***

## Supplemental information

# Comprehensive assessment of on- and off-target mutagenesis via lipid nanoparticle delivery of CRISPR-Cas9 genome editing

**Youichi Naoe, Naoko Fujimoto, Yukimasa Makita, Dongyang Li, Naoto Inukai, and Akitsu Hotta**

# Figure S1

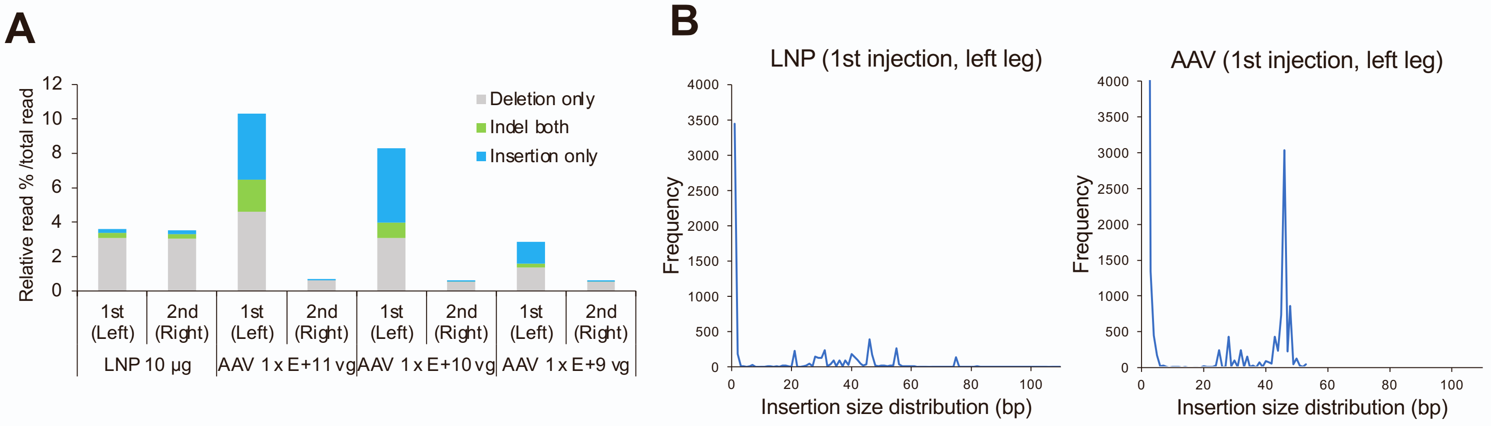


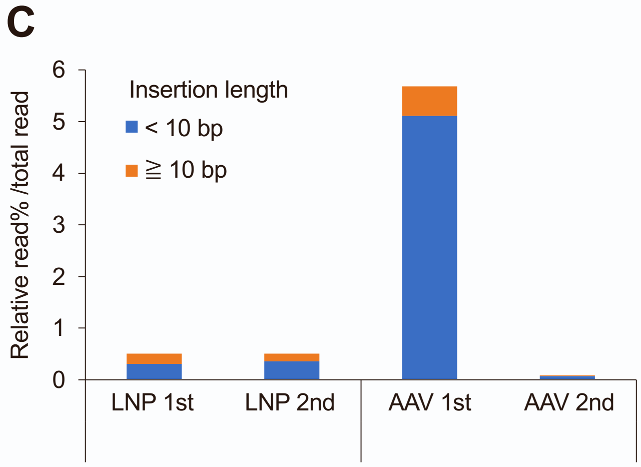


D

| | LNP | | AAV |
|---|---|---|---|
| | 1st | 2nd | 1st |
| Total insertion reads (≧ 10 bp) | 2,942 | 2,315 | 8,726 |
| *DMD* exon 45 reads | 1,579 | 1,332 | 5,091 |
| Inverted *DMD* exon 45 reads | 1,576 | 1,324 | 5,076 |
| Percentage of inverted reads at *DMD* exon 45 | 99.8% | 99.4% | 99.7% |

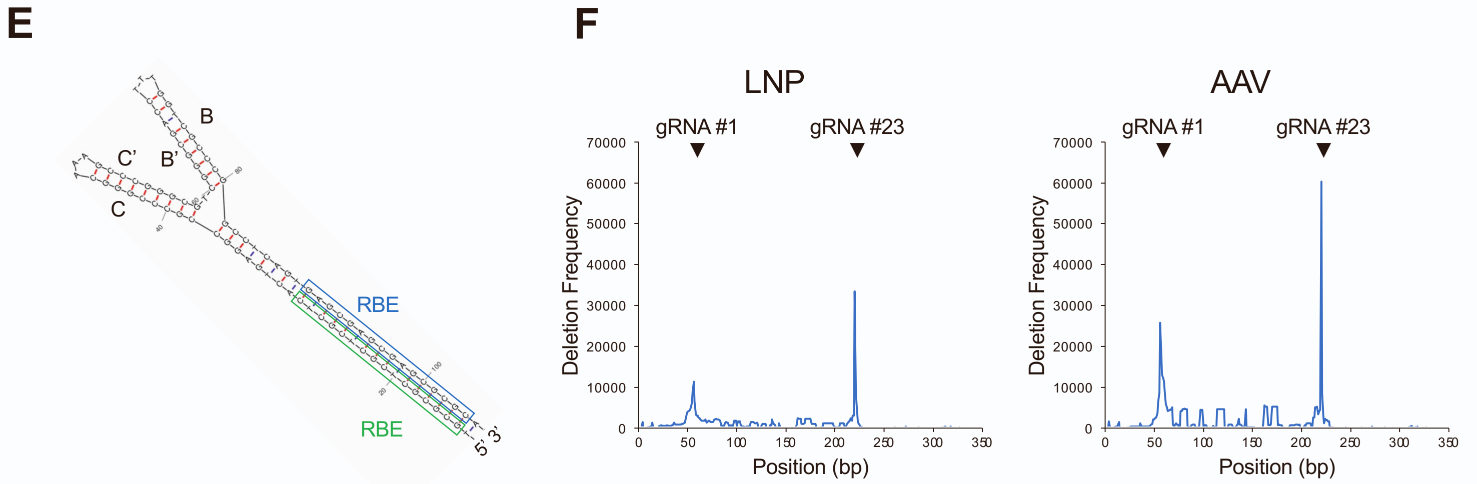


**Figure S1: Amplicon-seq analysis of on-target site in CAG-Luc2-hDMD Ex45 knock-in reporter mice.**

A. Comparison of indel event proportions between LNP and AAV at various doses.

B. Size distribution of the insertion sequences. CAG-Luc2-hDMD Ex45 knock-in mice were administered with LNP-CRISPR (10 µg) or AAV9-CRISPR ($1 \times 10^{11}$ v.g.). The first injection was on the left gastrocnemius (GC) muscle, and the second injection was on the right GC muscle. After genome DNA extraction, the on-target region of the DMD exon 45 was PCR amplified and analyzed by amplicon-seq. Among the detected indel patterns, only the insertion sequences were extracted, and their sizes are plotted.

C. Among the insertion sequences, the ratio of sequences longer than 10 bp or equal to 10 bp is indicated in orange, and shorter than 10 bp in blue.

D. Out of the detected human *DMD* exon 45 sequence from the insertion sequences, the percentage of inversion is indicated.

E. Schematic of DNA secondary structure of the ITR (inverted terminal repeat) region of the AAV vector backbone sequence. Rep binding elements (RBEs) and palindromic arms (B-B' and C-C') are indicated.

F. Deletion frequency at each position relative to the two gRNA target sites. Left: LNP-CRISPR-injected mice. Right: AAV-CRISPR-injected mice.

# Figure S2

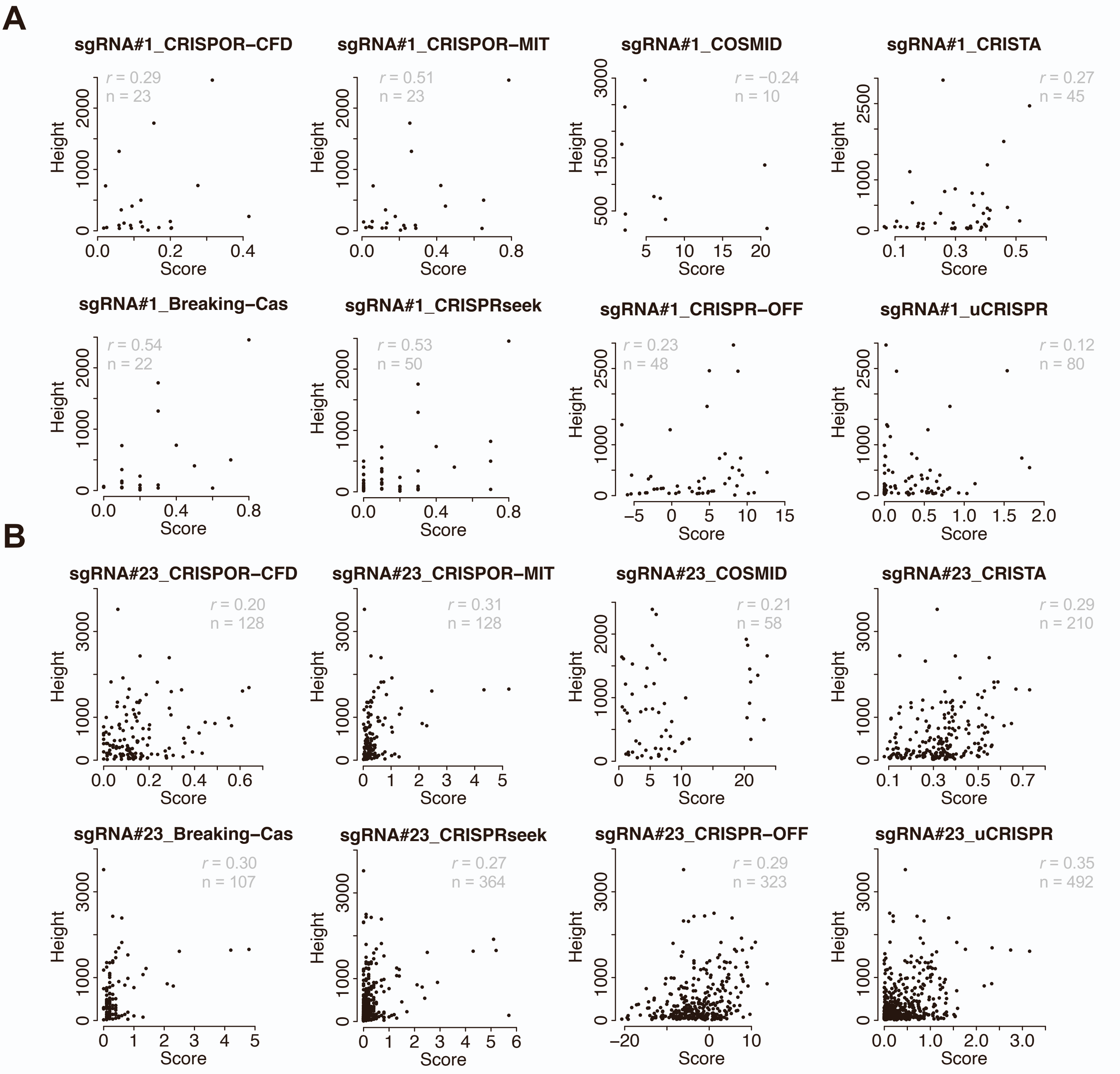


**Figure S2: Performance evaluation of *in silico* prediction tools for potential off-target binding/cleavage sites.**
A. The sequence of the gRNA #1 was provided to each *in silico* prediction tool, and potential off-target sites were obtained. After extracting the overlapping site with our CIRCLE-seq experimental results, the output specificity scores from the tools were plotted against the CIRCLE-seq peak heights, as an indicator of potential DNA cleavage activity *in vitro*. The number of overlapping sites is shown as “n”, and the correlation coefficient is shown as ”*r*". For CRISPOR, both the CFD Specificity score and the MIT Guide Specificity score were utilized for plotting.
B. Scattered plots of the tool specificity scores against the CIRCLE-seq peak heights by using the gRNA #23

# Figure S3

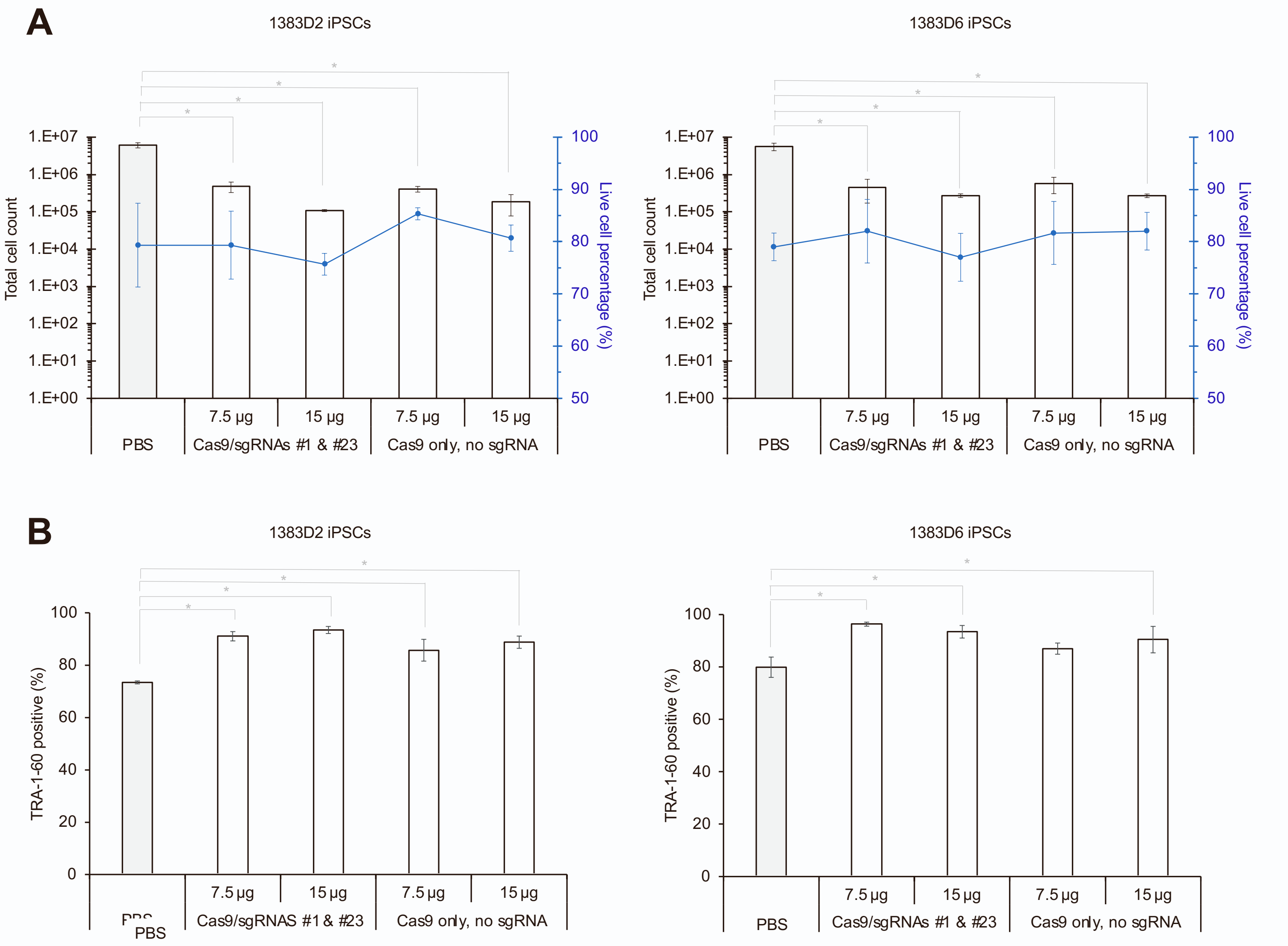


**Figure S3: Effects of LNP on cell viability and pluripotency in iPSCs.**
A. Effects of LNP on iPS cell viability. The left y-axis (black) shows total cell count and right y-axis (blue) shows live cell percentage, both presented as mean ± S.D. (n = 3 wells), and the x-axis represents the experimental conditions. Total RNA amount at two levels (7.5 µg and 15 µg) were used to treat iPSC in the presence or absence of gRNA, and the results were compared with the PBS group. The left panel shows iPSC line 1383D2, and the right panel shows 1383D6. Statistical significance was determined by one-way ANOVA followed by Dunnett's multiple comparison test versus PBS. * $p < 0.05$. No significant differences were observed for live cell percentage (right y-axis).
B. Effects of LNP treatment on pluripotency in iPSC lines 1383D2 and 1383D6. The y-axis indicates TRA-1-60 positive cell percentage of iPSCs assessed by flow cytometry shown as mean ± S.D. (n = 3 wells). Note that PBS treated iPS cells were overconfluent and therefore exhibited slightly lower TRA-1-60 positiveness. Statistical significance was determined by one-way ANOVA followed by Dunnett's multiple comparison test versus PBS. * $p < 0.05$.

# Figure S4

**A**

| iPSC line | Treated gRNA | Total seq. reads | Mapped reads | Mean depth |
|---|---|---|---|---|
| 1383D2 | - | 1,550,594,738 | 96.5% | 79.25 |
| | #1 | 1,389,839,194 | 96.6% | 71.03 |
| | #23 | 1,477,430,064 | 96.7% | 75.51 |
| | #1 + #23 | 1,555,112,436 | 96.6% | 79.48 |
| 1383D6 | - | 1,269,887,554 | 96.5% | 64.90 |
| | #1 | 1,284,051,876 | 96.5% | 65.63 |
| | #23 | 1,591,064,548 | 96.4% | 81.32 |
| | #1 + #23 | 1,205,774,840 | 96.5% | 61.63 |

**B**

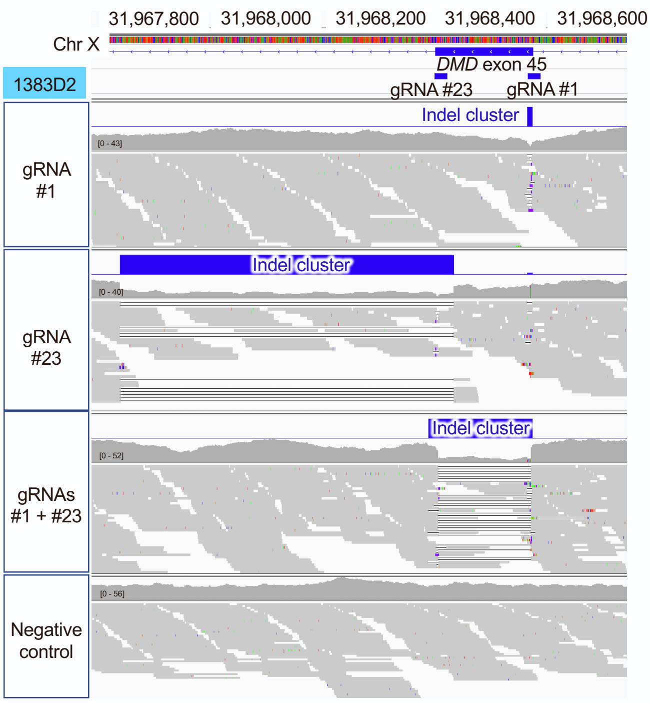


**C**

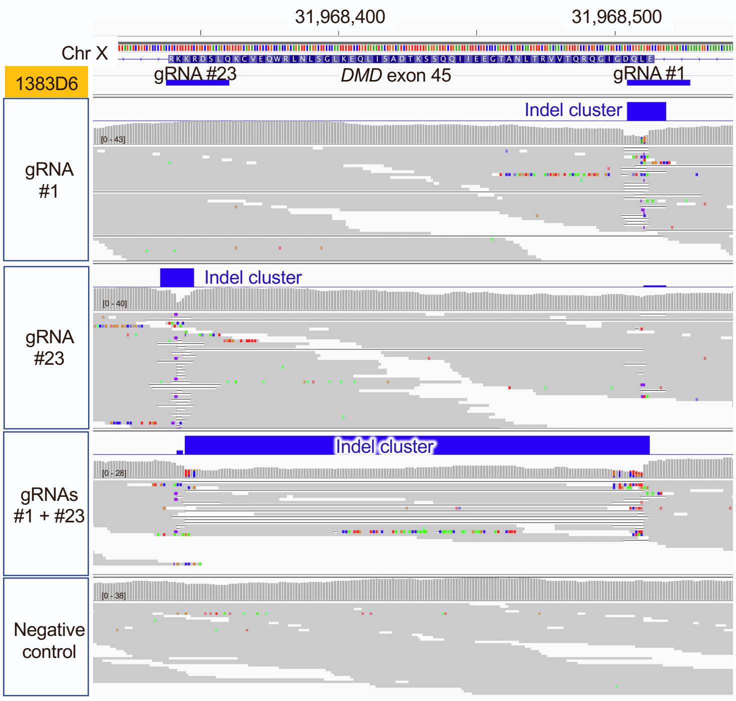


**Figure S4: Summary of whole genome sequencing analysis and on-target confirmation.**
A. Summary statistics of the sequencing and mapping results for human iPSCs treated with LNP-CRISPR.
B. Validation of the on-target genome editing from the WGS analysis. iPSC 1383D2 line was treated with LNP-CRISPR delivering gRNA #1 only, gRNA #23 only, or a combination of gRNAs #1 and #23. Mapped sequence reads at the *DMD* gene exon 45 region are shown in light gray bars at the bottom, coverage depths are shown in dark gray peaks at the middle, and the “indel clusters” detected *de novo* from the negative control (in Figure 3F) are shown in a blue bar graph at the top. For the negative control, only mapped sequence reads are shown.
C. Similar to above, the iPSC 1383D6 line was treated with LNP-CRISPR and analyzed by WGS.

# Figure S5

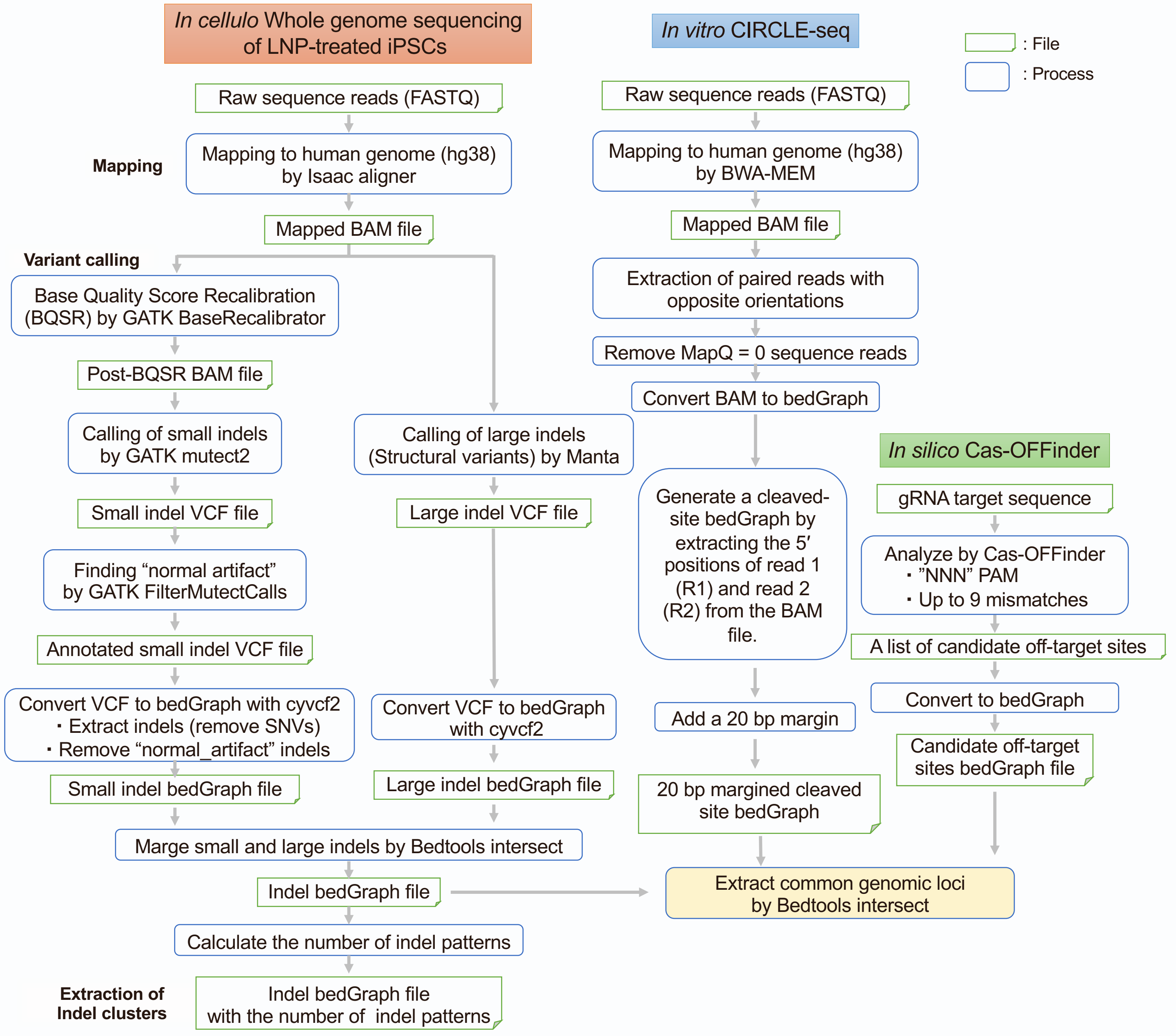


**Figure S5: The analytical flowchart of the data integration of WGS, CIRCLE-seq, and Cas-OFFinder data.**
File format is indicated by a light green rectangle with a folded corner, and process is indicated by a light blue rounded rectangle.

# Figure S6

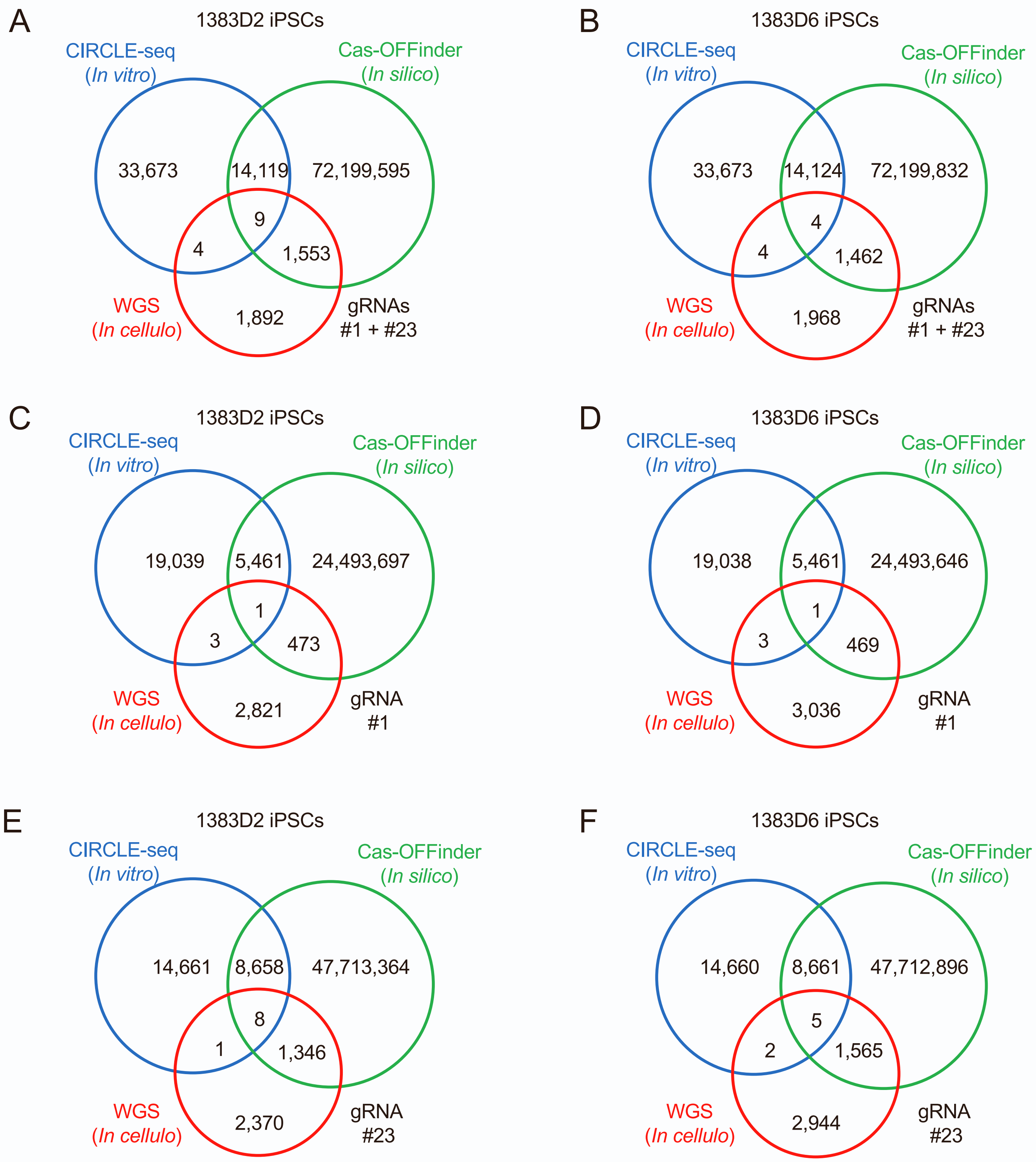


**Figure S6: Venn diagram of the combined off-target sites of WGS, CIRCLE-seq, and Cas-OFFinder analyses.** Related to Figure 4B, the number of overlapping sites is indicated in the Venn diagram. Left side (A, C, E): data from 1383D2 iPSCs, right side (B, D, F): data from 1383D6 iPSCs. Top row (A, B): WGS data with gRNA #1 and #23 treatment, middle row (C, D): WGS data with gRNA #1 only, bottom row (E, F): WGS data with gRNA #23 only. Notably, all the on-target sites were detected as common from the three analysis methods.

# Figure S7

## A

| Primer set ID (gRNA position) | Forward 5'-3' | Reverse 5'-3' | Annealing temp. (° C) | PCR product size (bp) |
|---|---|---|---|---|
| chrX:31968338-31968527 (On-target) | AGTGCCTTTCACCCTGCTTA | GGGCATGTCAGTTTCATAGGGA | 60 | 432 |
| chr1:17729009-17729031 | CGAGTGGGAGGAGATGAAATAGG | CTTGAACAAGTCAGGAGACCTCA | 60 | 447 |
| chr1:84361946-84361968 | CTGAAGGGAAGAGGAGCTTATGT | AAGACTCCAGGGGAGATTCTGT | 60 | 406 |
| chr3:67017674-67017696 | CTCACTTGGTGTTTGTGTAGCTG | AGTGCCCAGTAAAGTGTGGATAG | 60 | 489 |
| chr5:113937846-113937868 | GACAGAGCACTTATGTTTCAGGC | TGTTTCCCACCTGTGTTCCATAT | 60 | 492 |
| chr6:137734937-137734959 | ACAGGGAAAAGCCAAGCCTTAC | CCCATCACAAGGAGGAAGTAGG | 60 | 475 |
| chr7:140404449-140404471 | GGTAAGCGACAGGTGAACCA | CCCTGCCTTGTGACATCCTT | 60 | 484 |
| chr9:11687303-11687325 | GGATTCCCTCCCCAGACAAAC | CCATGGTACTGAGTGGGTGAA | 60 | 432 |
| chr14:67492431-67492453 | TGTGTTACTGTAAAGAGCTGCCT | TAGAAATCTCTAGGTGGCTTGGC | 63 | 408 |
| chr18:58827057-58827079 | TTTCCAGGGAAAGGAAACCCAG | AGAACTGTACACTCACCGCG | 63 | 460 |
| chr20:58378315-58378337 | ATGCCAATAAGGTGCTGGAC | TTTGCATTTCCTCAGCATCTACT | 60 | 400 |
| chrX:96288780-96288802 | AAAGGACATCTCACCGTTGTCT | TCTGAGTGGCAACTAAATGGGTC | 60 | 400 |

## B

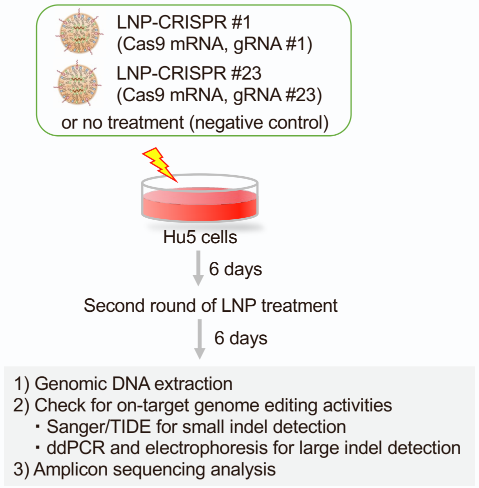


## C

| Small indel % by TIDE assay | Hu5 | |
|---|---|---|
| gRNA | #1 | #23 |
| Indel (%) | 63.9 | 67.7 |

## D

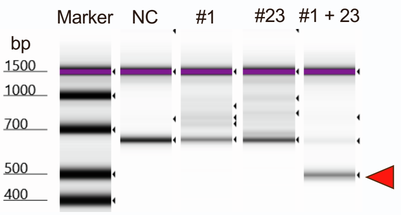


**Figure S7: Primer list for amplicon sequencing and and preparation of human myogenic Hu5 cells.**
A. Primer pairs used to amplify the candidate off-target loci, related to Figure 4D. The on-target site highlighted in light beige was incorporated as positive control. “Annealing temp. (℃)” indicates the PCR annealing temperature used for amplicon sequencing library preparation to minimize non-specific amplifications.
B. Experimental workflow for genome editing in Hu5 cells using LNP-mediated CRISPR-Cas9 delivery.
C. Small indel frequencies at the on-target site determined by TIDE analysis of Sanger sequencing data in Hu5 cells following LNP-CRISPR treatment with gRNA #1 or gRNA #23.
D. Representative TapeStation electrophoresis image of PCR-amplified on-target regions in Hu5 cells. NC indicates untreated negative control. The red arrow indicates the deletion fragment generated between the two CRISPR target sites.

# Figure S8

**A**

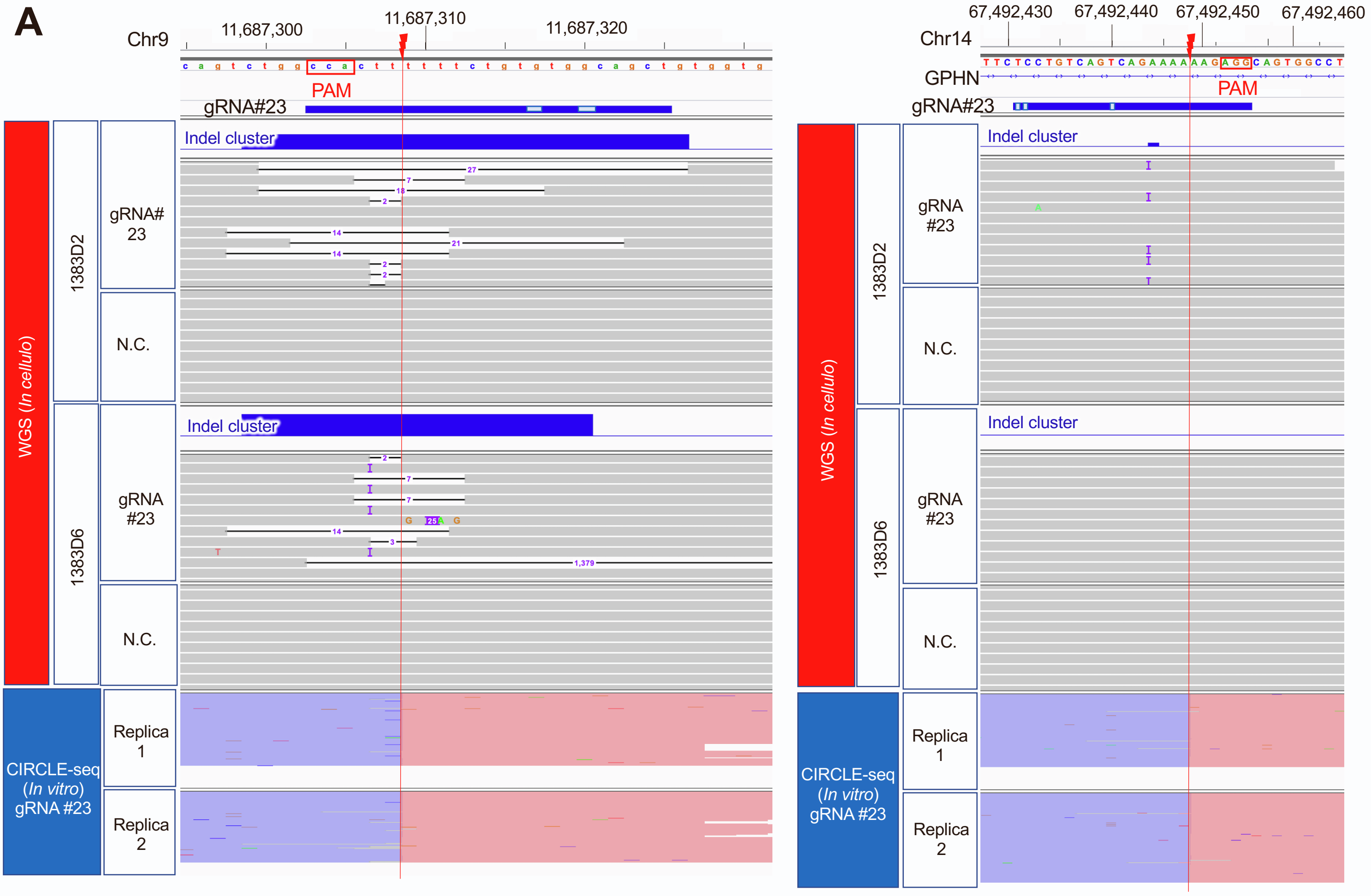


**B**

| | | WGS | | | | | | | CIRCLE-seq | | | | |
|---|---|---|---|---|---|---|---|---|---|---|---|---|---|
| gRNA-independent off-target | | WGS_off-target_site | Allele Frac | Indel pattern | Genome_edited | | NC | | Peak height | Used gRNA | Repeat element | Problematic region | Mappability |
| | | | | | Mean_MapQ | No._Reads | Mean_MapQ | No._Reads | | | | | |
| 1383D2 iPSC | gRNA #1 | chr4_120645206_120645206 | 0.061 | 1 | 53.87 | 94 | 54.37 | 99 | 0, 18 | #1 | LINE | Yes | 0 |
| | | chr8_51696703_51696703 | 0.046 | 1 | 44.92 | 148 | 45.15 | 170 | 0, 30 | #1 | - | Yes | 0.71 |
| | | chr16_34581526_34581528 | 0.003 | 1 | 28.86 | 10602 | 29.39 | 12094 | 65, 109 | #1 | - | Yes | 0 |
| | #23 | chr4_49637782_49637784 | 0.153 | 1 | 22.36 | 1168 | 23.59 | 1310 | 2, 23 | #23 | Satellite | Yes | 0.08 |
| | #1 + #23 | chr4_49637782_49637784 | 0.086 | 1 | 22.17 | 1191 | 23.59 | 1310 | 2, 23 | #23 | Satellite | Yes | 0.08 |
| | | chr9_63664629_63664629 | 0.056 | 1 | 38.69 | 140 | 40.66 | 109 | 0, 11 | #1 | Simple repeat | Yes | 0.13 |
| | | chr22_11813420_11813420 | 0.014 | 1 | 12.7 | 5018 | 12.08 | 5057 | 11, 0 | #23 | Simple repeat | Yes | 0 |
| | | chr22_26210002_26210002 | 0.160 | 1 | 55.22 | 118 | 57.03 | 128 | 0, 10 | #23 | Low complexity | Yes | 0.67 |
| 1383D6 iPSC | #1 | chr9_63664618_63664627 | 0.075 | 1 | 35.94 | 118 | 40.29 | 105 | 0, 11 | #1 | Simple repeat | Yes | 0.0375 |
| | | chr16_46387236_46387257 | 0.003 | 1 | 21.32 | 27246 | 21.59 | 26199 | 0, 22 | #1 | - | Yes | 0 |
| | | chr21_9248177_9248178 | 0.016 | 1 | 52.3 | 1167 | 51.68 | 1137 | 0, 13 | #1 | - | Yes | 0.72916 |
| | #23 | chr1_153505473_153505473 | 0.036 | 1 | 60 | 167 | 60 | 100 | 21, 0 | #23 | SINE | No | 0.29 |
| | | chr14_52845949_52846136 | 0.021 | 1 | 56.58 | 205 | 57.37 | 137 | 0, 19 | #23 | SINE | Partially Yes | 0.154 |
| | #1 + #23 | chr2_117780613_117780614 | 0.079 | 1 | 57.77 | 95 | 58.1 | 103 | 0, 34 | #23 | LINE | Yes | 0 |
| | | chr7_61048180_61048180 | 0.023 | 1 | 57.86 | 355 | 56.55 | 347 | 0, 28 | #1 | - | Yes | 0.29 |
| | | chr10_124227297_124227348 | 0.033 | 1 | 52.76 | 259 | 54.25 | 295 | 28, 0 | #23 | - | Yes | 0.664 |
| | | chrY_20278947_20278947 | 0.045 | 1 | 54.88 | 159 | 55.94 | 174 | 0, 66 | #1 | LTR | Yes | 0 |

| On-target | | WGS_off-target_site | Allele Frac | Indel pattern | Genome_edited | | NC | | Peak height | Used gRNA | Repeat element | Problematic region | Mappability |
|---|---|---|---|---|---|---|---|---|---|---|---|---|---|
| | | | | | Mean MapQ | No. Reads | Mean MapQ | No. Reads | | | | | |
| 1383D2 iPSC | #1 | chrX_31968504_31968512 | 1.810 | 3 | 60 | 45 | 60 | 62 | 1562, 5245 | #1 | - | No | 1 |
| | #23 | chrX_31967773_31968371 | 1.688 | 5 | 59.31 | 87 | 60 | 221 | 2023, 3129 | #23 | - | Partially Yes | 1 |
| | #1 + #23 | chrX_31968326_31968512 | 1.522 | 5 | 58.04 | 92 | 60 | 118 | 2023, 3129 | #23 | - | No | 1 |
| | | chrX_31968326_31968512 | 1.522 | 5 | 58.04 | 92 | 60 | 118 | 1562, 5245 | #1 | - | No | 1 |
| 1383D6 iPSC | #1 | chrX_31968504_31968518 | 1.599 | 4 | 60 | 68 | 60 | 62 | 1562, 5245 | #1 | - | No | 1 |
| | #23 | chrX_31968335_31968347 | 1.064 | 7 | 59.96 | 61 | 60 | 60 | 2023, 3129 | #23 | - | No | 1 |
| | #1 + #23 | chrX_31968341_31968343 | 0.368 | 2 | 58.28 | 35 | 60 | 58 | 2023, 3129 | #23 | - | No | 1 |
| | | chrX_31968344_31968512 | 1.656 | 4 | 58.88 | 54 | 60 | 96 | 2023, 3129 | #23 | - | No | 1 |
| | | chrX_31968344_31968512 | 1.656 | 4 | 58.88 | 54 | 60 | 96 | 1562, 5245 | #1 | - | No | 1 |

**Figure S8: Detailed analysis of the off-target sites detected.**

A. Two representative off-target sites detected from the comprehensive analysis. Left: chr9:11687303-11687325 in iPSCs 1383D2 and 1383D6 treated with gRNA #23. Right: chr14:67492431-67492453 in iPSC 1383D2 treated with gRNA #23. The mismatch positions on the gRNA sequence are shown in light blue. The red lightning mark indicates a Cas9 cleavage site. The CIRCLE-seq reads shown in blue and red represent the minus and plus strands, respectively, and the junction indicates the DNA cleavage site.

B. Putative gRNA-independent off-target sites that were detected by experimental WGS and CIRCLE-seq, but not by *in silico* analysis (upper). Repeat element (RepeatMasker), Problematic regions, and Mappability are annotated based on the UCSC Genome browser. For the Mean MapQ and Mappability, the deeper color indicates a higher value. A table similar to the one above for the on-target sites (lower).
