# Supplementary material for: Comprehensive assessment of on- and off-target mutagenesis via lipid nanoparticle delivery of CRISPR-Cas9 genome editing
Source: Mol Ther Nucleic Acids. 2026 May 20;37(2):102958. doi: 10.1016/j.omtn.2026.102958 (PMC13267559; doi:10.1016/j.omtn.2026.102958)
Supplement: Document S2. Article plus supplemental information [file mmc2.pdf]

Molecular Therapy
Nucleic Acids
Original Article

Comprehensive assessment of on- and off-target mutagenesis via lipid nanoparticle delivery of CRISPR-Cas9 genome editing

Youichi Naoe,[1,2] Naoko Fujimoto,[1,2] Yukimasa Makita,[2,3,4] Dongyang Li,[1,2] Naoto Inukai,[2,3] and Akitsu Hotta[1,2]

[1]Department of Clinical Application, Center for IPS Cell Research and Application (CiRA), Kyoto University, Kyoto, Japan; [2]Takeda-CiRA Joint Program for IPS Cell Applications (T-CiRA), Fujisawa, Kanagawa, Japan; [3]Target Validation Sciences, Takeda Pharmaceutical Company Limited, Fujisawa, Kanagawa, Japan

Ensuring the safety of CRISPR-Cas9 genome editing requires comprehensive assessment of off-target mutagenesis, particularly for therapeutic applications under regulatory review. DNA-free lipid nanoparticle (LNP) delivery is expected to minimize insertional risks compared to adeno-associated virus (AAV) vectors, but experimental validation has been limited. Here, on-target amplicon sequencing in mouse muscle demonstrated insertions largely derived from host genomic sequences, with no detectable AAV or transgene integration. Benchmarking 13 *in silico* prediction tools identified Cas-OFFinder as the most sensitive, though precision remained low, and correlation with *in vitro* CIRCLE-seq data was modest. To strengthen off-target detection, we employed karyotypically normal human iPSCs and developed an "indel cluster" method using high-depth whole-genome sequencing, enabling discrimination between clustered genome editing-induced indels and background variants. Integration of *in silico* predictions, CIRCLE-seq cleavage sites, and *in cellulo* WGS clusters yielded 11 high-confidence off-target candidates, predominantly associated with one gRNA. While sensitivity was maximized, reproducibility across conditions remained limited, and many candidate sites overlapped repetitive or low-mappability regions. Our multilayered framework demonstrates both the utility and current limitations of off-target risk assessment, providing a practical foundation for the safety evaluation of genome editing therapies.

INTRODUCTION

Due to its versatility, the CRISPR-Cas9 system is a promising modality for treating intractable diseases caused by genetic mutations.[1] Duchenne muscular dystrophy (DMD) is a severe hereditary disease caused by a reading frame shift in the *dystrophin* gene, leading to the loss of the dystrophin protein and progressive muscle degeneration, which can ultimately cause premature death from heart or respiratory failure. CRISPR genome editing has been proposed as a strategy to induce genomic exon skipping, thereby restoring the reading frame of the *DMD* gene and allowing the expression of in-frame dystrophin protein.[2–5] Since genome editing outcomes are irreversible, and unintended mutations in tumor suppressor genes may pose oncogenic risks, a key safety aspect associated with genome editing therapy strategies is minimizing and monitoring unintended DNA alterations, both at the on-target and off-target sites.

The genome editing activity depends on both the magnitude and duration of exposure to the Cas9 and guide RNA (gRNA) complex; hence, the delivery modality is a key consideration for effectiveness and safety. For this, adeno-associated virus (AAV) vectors, lipid nanoparticles (LNPs), and other platforms have been utilized to deliver Cas9 and gRNA *in vivo* to treat DMD.[3,4,6] However, in the case of AAV-CRISPR, unintended genomic integration of vector DNA fragments has been reported.[7–9] The single-stranded genomic DNA of AAV vectors persists long-term within the nucleus as episomes, posing a risk of integration into the genome when double-strand breaks are induced. LNPs serve as RNA-based delivery vehicles without DNA, and even if cytoplasmic RNA were to be reverse-transcribed, it is unlikely to affect genomic DNA in the nucleus; however, there is little experimental validation for this.

Regarding off-target sites, the CRISPR-Cas9 system tolerates a few base pair mismatches between gRNA and target DNA, which sometimes causes undesired DNA cleavage.[10,11] Several computational tools have been reported to predict potential off-target sites based on sequence similarity to gRNA targets.[12] However, there is variation in the results of off-target assessment among these tools, and there are no specific guidelines on which tools are most appropriate for clinical development and prediction. This variation makes it difficult to predict or evaluate bona fide off-target sites.

CRISPR-Cas9/gRNA introduces DNA breaks, which activate the host's DNA repair mechanisms and induce mutations in the DNA sequence. The off-target mutagenesis risk of CRISPR-Cas9 is often evaluated in immortalized cancer cell lines, such as HEK293T cells.

Received 5 October 2025; accepted 15 May 2026;
https://doi.org/10.1016/j.omtn.2026.102958.

[4]Present address: Axcelead Drug Discovery Partners, Inc., Fujisawa, Kanagawa, Japan

Correspondence: Akitsu Hotta, Room II-502, 53 Shogoin-Kawahara-cho, Sakyo-ku, Kyoto, Japan.
E-mail: akitsu.hotta@cira.kyoto-u.ac.jp

© 2026 The Author(s).
Published by Elsevier Inc. on behalf of The American Society of Gene and Cell Therapy.
This is an open access article under the CC BY license (http://creativecommons.org/licenses/by/4.0/).

However, these cancer cells often harbor abnormalities in DNA repair pathways, which may compromise the accuracy of risk assessment. Therefore, the use of karyotypically normal yet indefinitely expandable iPSCs (induced pluripotent stem cells) provides a reliable platform for evaluating off-target mutagenesis.

In silico tools are simple and inexpensive, but whether DNA breaks actually occur at the predicted sites needs to be experimentally verified. *In vitro* methods that test for DNA breaks (such as CIRCLE-seq, Digenome-seq, SITE-seq, CHANGE-seq, and DIG-seq) are less biased but cannot determine if mutations are induced at those sites.[13–17] *In cellulo* whole-genome sequencing (WGS) can comprehensively confirm the location of DNA mutations, but it is necessary to distinguish between mutations that occur spontaneously in culture and those induced by genome editing. In the context of these challenges, integrated analysis that combines at least one *in silico* method and one *in vitro* or *in cellulo* method is recommended to nominate a list of candidate off-target sites as part of preclinical evaluations.[18,19]

Here, we first investigated the on-target amplicon sequencing analysis of the LNP-delivered CRISPR-Cas9 system and found that the insertional event was less frequent compared to AAV vector delivery. Next, we performed a cross-sectional evaluation of a total of 13 software programs to predict off-target sites *in silico*. We evaluated sensitivity and precision to identify the most suitable software, which is less likely to overlook bona fide off-target sites. Third, we treated two human iPSC lines with LNP-CRISPR at a high dose and performed WGS to extract *de novo* indels relative to the untreated control iPSCs. Lastly, we combined the *in silico* prediction to list potential gRNA binding sites, *in vitro* CIRCLE-seq analysis to search for potential DNA cleavage sites, and *in cellulo* WGS of iPSCs to identify "indel cluster" regions that are potentially caused by Cas9/gRNA cleavage. A risk assessment was performed to determine whether the candidate off-target sites detected by the above methods overlapped with the gene-coding regions and whether the nearby genes are associated with oncogenesis. Our findings provide a comprehensive and efficient framework for identifying off-target mutational risks associated with the CRISPR-Cas9 genome editing system and for developing safe treatments for genetic disorders.

RESULTS

On-target indel analysis by LNP and AAV delivery of dual-CRISPR-Cas9 in mice

To restore the protein reading frame of the dystrophin gene by genomic exon skipping, our group adopted dual gRNAs #1 and #23 that target the splicing acceptor and donor of human *DMD* exon 45, respectively (Figure 1A).[20] We first performed detailed on-target amplicon sequencing (amplicon-seq) to evaluate the safety of the dual gRNA CRISPR system by comparing LNP and AAV vector delivery in mouse skeletal muscles (Figure 1B). LNP or AAV vectors packaged with dual gRNAs were injected into Luc reporter mice (CAG-Luc2-hDMD Ex45 knock-in mice), which can monitor genomic exon skipping activity noninvasively *in vivo* as luciferase activity.[21] We first injected LNP or AAV vectors into the left leg (gastrocnemius muscles) of Luc reporter mice and, 28 days later, injected the same dose into the contralateral (right) leg to assess the effect of repeated administration. We selected a 28-day interval because we previously observed an elevation of anti-Cas9 antibody levels in plasma of AAV-injected mice at days 30 and 43 post-injection, but not LNP-injected ones.[21] Then, on-target amplicon-seq analyses of genomic DNA from muscle tissues were performed 28 days after the second injection. By using the CRISPResso software, we measured the rate of insertion and deletion (indel) for each administration (Figure 1C).[22] For the AAV vector, the indel efficiency with the first administration was 10.29%, but it dropped sharply to 0.68% with the second administration. On the other hand, for LNP, both the first and the second administrations showed almost the same indel read ratio, 3.60% and 3.55%, respectively. This result suggests that the LNP can be administered repeatedly and may retain a comparable indel ratio in subsequent administrations.

Since it has been reported that AAV delivery of CRISPR-Cas9 results in a high risk of vector fragment integrations, we also investigated the inserted sequences at the on-target site (Figure 1D).[7–9,23] LNP showed a relatively small insertion ratio of 0.51% for the first and the second administrations (including the "indel both" condition). On the other hand, the AAV (1st injection) showed as high as 5.69% of sequence reads containing insertion events. This higher proportion of insertion events observed with AAV compared to LNP was also confirmed at different doses (1×10^{10} v.g. and 1×10^{9} v.g.; Figure S1A). Then, we examined the size distribution of the inserted sequences (Figures S1B and S1C). In both LNP and AAV conditions, the majority of insertions were shorter than 10 bp (59.3% and 89.8% within the total insertion events, respectively). Since such short sequences are difficult to uniquely locate to the origin, we focused on the inserted sequences longer than 10 bp and performed a BLAST sequence homology search (Figure 1D). For LNP, more than half (52.8% for 1st injection and 53.8% for 2nd injection) of the inserted sequences matched a part of the human *DMD* exon 45 sequence. We also detected mouse repeat sequences (including LINE, simple repeat, etc.) and other mouse genomic sequences. Transgene-derived sequences (Cas9 mRNA and gRNAs) were barely detected in the LNP-treated samples. In contrast, in the case of AAV treatment, 37.0% of the insertions were part of the AAV vector sequence, in addition to 58.1% of the *DMD* exon 45 sequences. Regarding the inserted sequences derived from the *DMD* exon 45, we found that most of the inserted sequences were inverted at the two gRNA cleavage sites (Figures 1E and S1D). Among the inserted sequences of the AAV vector, almost all insertions (99.97%) were mapped onto the inverted terminal repeat (ITR) region (Figure 1F). More specifically, the inserted sequences were concentrated on the two Rep-binding elements (RBE) on the ITR region, which are known to form stable double-stranded DNA as a secondary stem structure (Figure S1E). We also investigated the integration site of the AAV vector sequences, and they were located at the gRNA target sites (Figure 1G). Although

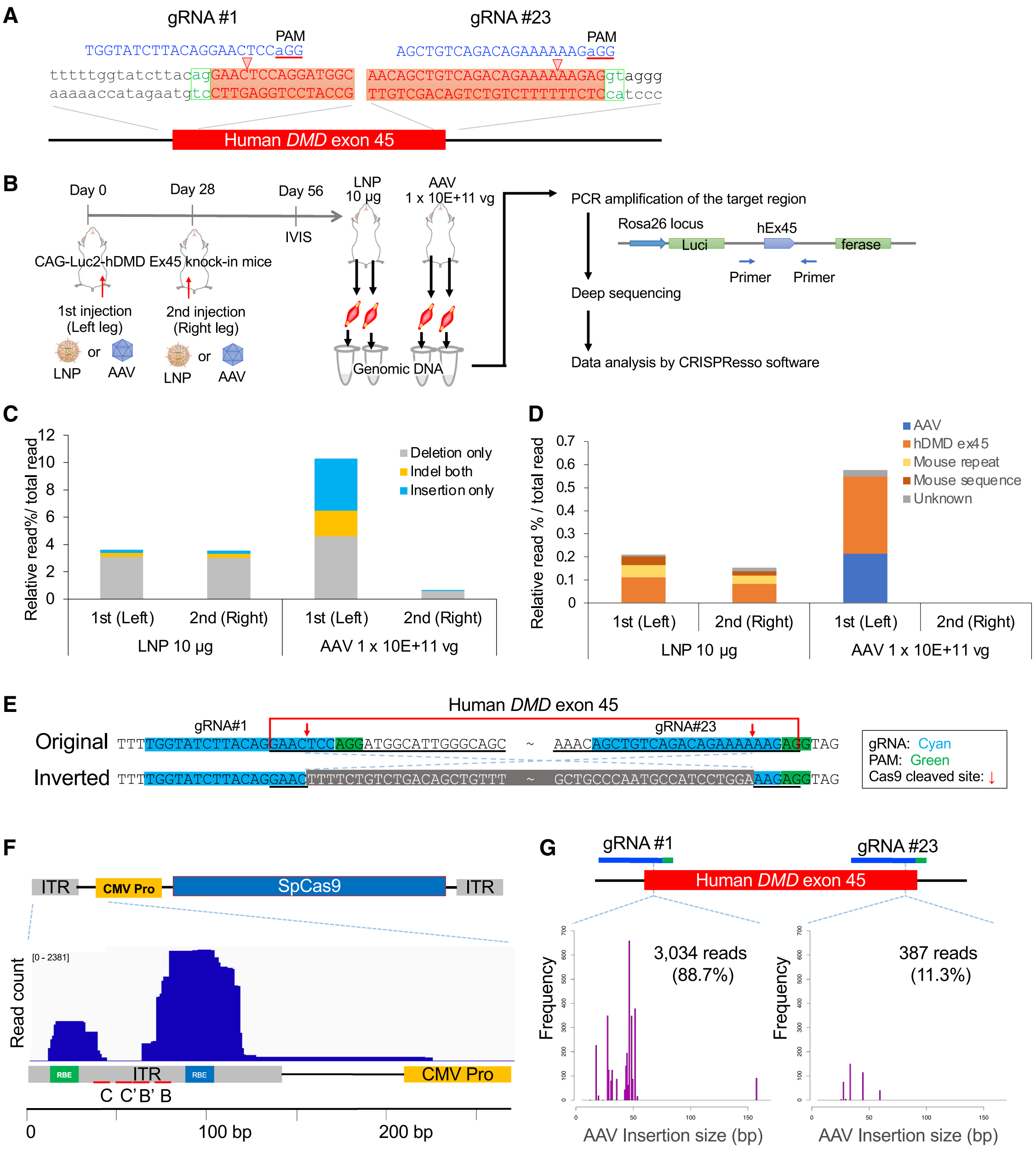

Figure 1. Analysis of on-target insertion and deletion pattern by amplicon sequencing after LNP or AAV delivery of CRISPR-Cas9 and dual gRNAs

(A) To induce exon 45 skipping in the human *DMD* gene, gRNA #1 targets the splicing acceptor site, and gRNA #23 targets the splicing donor site.

(B) Overview of amplicon-seq analysis. LNP-CRISPR (10 μg of RNA) or AAV-CRISPR (1 $\times$ 10^{11} vg) encoding the dual gRNAs (#1 and #23) were injected into the hindlimb of Luc reporter (CAG-Luc2-hDMD Ex45 knock-in) mice (4 mice per group) twice on day 0 (left leg) and day 28 (right leg). On day 56, skeletal muscle tissues were isolated, and amplicon sequencing was performed.

(C) On-target genome editing efficiency. Among the mapped sequencing reads, the percentages of deletion only (gray), insertion only (blue), and both insertion and deletion detected in one sequencing read (yellow) are shown as means from the four mice per group.

(D) Among the insertion sequences longer than 10 bp, the origin of the sequences was classified by BLAST search. AAV vector-derived insertion sequence (mainly ITR region) is indicated in blue, human *DMD* exon 45 sequence is in orange, mouse repeat sequence (defined by RepeatMasker) is in yellow, mouse genomic sequence (non-repeat region) is in brown, and other unknown (unidentifiable) sequences are in gray. Data represent means from four mice per group.

(E) The majority of the human *DMD* exon 45 sequence in the insertion sequence was an inversion between the two gRNA cutting sites (red arrowheads indicate Cas9 cleavage sites).

(F) AAV-derived sequences were mainly mapped to the ITR region. Top: schematic of the AAV vector encoding the front half of the SpCas9 cDNA. ITR: inverted terminal repeat (gray box); CMV Pro: cytomegalovirus promoter (yellow box); RBE: Rep-binding element. Bottom: read counts mapped to the AAV ITR region (blue histogram).

(G) The location and size distribution of the inserted AAV sequences.

we observed higher insertion events at the gRNA #1 site, the deletion events were more frequent at the gRNA #23 site (Figure S1F).

Cross-sectional analysis of *in silico* off-target prediction tools

To nominate candidate off-target sites in the human genome based on sequence homology, *in silico* prediction software is often utilized. We conducted a benchmarking analysis of 13 off-target prediction tools using our gRNA sequences and compared them with our CIRCLE-seq data.[21] For *in silico* tools, we utilized a previous benchmarking study (iGWOS) and WeReview repository to gather 14 representative *in silico* tools that predict SpCas9 off-target sites, based on sequence alignment (Cas-OFFinder, CHOPCHOP, GT-Scan, CRISPRdirect, Off-Spotter, Cas-Designer, GuideScan), hypothesis-driven (CRISPRseek, COSMID, Breaking-Cas, CRISPOR), machine learning (CRISTA), and binding energy (CRISPRoff, uCRISPR), respectively.[12,24–38] Then, we calculated potential off-target sites for gRNA #1 and gRNA #23 by using the default settings of PAM spectrum and mismatch base pair allowance (Figure 2A). uCRISPR is not shown in Figure 2A because it does not generate a list of potential off-target sites; instead, it provides sequence-specific scoring.

Utilizing the CIRCLE-seq cleaved sites (81 and 493 sites for gRNAs #1 and #23, respectively) as a benchmark, we first verified how well each tool detected the sites (Figure 2B). CRISTA, CRISPRdirect, CRISPRoff, Cas-OFFinder, and CRISPRseek cover almost all experimentally detected sites. On the other hand, those tools provided an overwhelming number of sites that were not detected by CRICLE-seq (Figure 2C). In other words, those tools produced a high number of false-positive sites. We plotted the sensitivity and precision of each tool using the CIRCLE-seq data as the reference set (Figure 2D). While CHOPCHOP, GuideScan, and GT-Scan exhibit high precision but low sensitivity, Cas-OFFinder, CRISPRoff, and CRISPRseek achieve high sensitivity at the cost of low precision. None of the *in silico* tools achieved both high sensitivity and high precision.

We compared the off-target scores provided by several *in silico* prediction tools with the cleavage activity measured by CIRCLE-seq, using peak height (i.e., the number of sequencing reads) as a proxy for cutting frequency. We observed limited correlation between predicted scores and experimentally determined cleavage activity (Figures S2A and S2B). In addition, the correlations were inconsistent between the two gRNAs. For example, Breaking-Cas showed the highest correlation coefficient of 0.54 with gRNA #1, but the correlation dropped to 0.30 with gRNA #23. Conversely, uCRISPR exhibited a modest correlation of 0.35 with gRNA #23, but only 0.12 with gRNA #1. These results suggest that current computational scoring schemes do not reliably reflect the quantitative extent of nuclease activity at off-target sites.

For clinical translation, sensitivity is of primary importance for *in silico* tools to minimize the risk of missing true off-target events, as undetected mutations in critical genes could have severe safety implications. The low precision of *in silico* prediction tools can be compensated by incorporating experimental datasets such as CIRCLE-seq, which provide unbiased *in vitro* detection of off-target cleavage sites.[13] Therefore, we re-visited the high-sensitivity tools, like Cas-OFFinder and CRISPRseek, by unifying the search space as a fully degenerate PAM (5′-NNN-3′) and allowing up to nine base pair mismatches. Since all the tools returned identical sites under matched parameters (Figure 2D, shown as diamonds), we chose to use Cas-OFFinder for the rest of the study because it is executable in a local environment with a simple interface.

WGS for comprehensive off-target analysis

In addition to *in silico* prediction tools and *in vitro* DNA cleavage assays, we evaluated whether genomic alterations are actually induced within cells by performing WGS in human cultured cells. Immortalized cells, such as cancer-derived cell lines, generally exhibit abnormal karyotypes and dysregulated DNA repair pathways. Consequently, the risk of off-target mutagenesis may not be faithfully assessed using such models. To address this limitation, we utilized induced pluripotent stem cells (iPSCs) derived from a healthy donor, which maintain normal karyotypes and lack oncogenic mutations in DNA repair pathways. To capture *in cellulo* off-target profiles that more accurately reflect mutagenesis outcomes under physiological DNA repair conditions, we performed WGS of two isogenic iPSC lines (1383D2 and 1383D6), both established from the same healthy donor, to confirm reproducibility in the same genomic context (Figures 3A and S4A). Furthermore, to investigate the effect of individual gRNA, we inoculated the iPSCs with LNP-CRISPRs under different conditions: gRNA #1 alone, gRNA #23 alone, or the combination of gRNA #1 and #23. To maximize the sensitivity for detecting off-target editing, we treated iPSC lines with high doses of LNP-CRISPRs to achieve saturating on-target editing (Figures 3B–3D). In single gRNA-treated samples, on-target indel frequencies were confirmed by Sanger sequencing and TIDE analysis, yielding 84.3% and 73.0% for the 1383D2 line and 83.7% and 67.7% for the 1383D6 line with gRNA #1 and gRNA #23, respectively (Figure 3B). In dual gRNA-treated samples, large deletions were quantified by ddPCR copy number analysis, with indel ratios of 96.6% and 89.6% for the 1383D2 line (gRNA #1 and #23 probes, respectively), and 97.6% and 80.9% for the 1383D6 line (Figure 3C). Amplicon electrophoresis further confirmed a ∼150 bp deletion corresponding to the region between gRNA #1 and gRNA #23 in dual gRNA-treated samples (Figure 3D). To assess the cytotoxicity of iPSCs treated with LNP-CRISPR, we measured cell counts in iPSC lines 1383D2 and 1383D6 after LNP treatment. Regardless of whether the LNP contains gRNA or not, LNP-treated groups showed significantly lower cell counts than the PBS control group, although no significant differences were observed in the percentage of live cells (Figure S3A). In addition, all LNP-treated groups exhibited more than 80% of TRA-1-60 positive cells (Figure S3B). These results suggest that high-dose LNP treatment affects cell viability but has minimal effect on iPSC pluripotency.

Having validated saturating on-target editing, we next performed high-depth WGS, achieving mean coverage depths of 71–79× for the 1383D2 line and 61–81× for the 1383D6 line (Figure S4A). To

A

Method	Mismatch allowance	No. of sites for gRNA #1	No. of sites for gRNA #23	Default PAM	Bulge	Specificity score
CIRCLE-seq	9	81	493	NNN	No	Yes
Cas-Designer	2	1	4	NGG	Yes	No
GuideScan	3	9	46	NRG	No	No
GT-Scan	3	9	46	NRG	No	No
CHOPCHOP	3	10	44	NGG	No	No
COSMID	3	69	193	NGG	Yes	Yes
Breaking-Cas	4	157	473	NGG	No	Yes
Off-Spotter	5	340	850	NGG	No	No
CRISPOR	5	852	1,864	NRG, NGA	No	Yes
CRISTA	13	994	2,794	NRR	Yes	Yes
CRISPRdirect	4	2,097	5,722	NGG	Yes	No
CRISPRoff	6	32,811	107,796	NRG, NGA	No	Yes
Cas-OFFinder	9	1,217,346	2,106,236	NGG	No	No
CRISPRseek	9	2,943,569	5,922,421	NGG, NAG	No	Yes

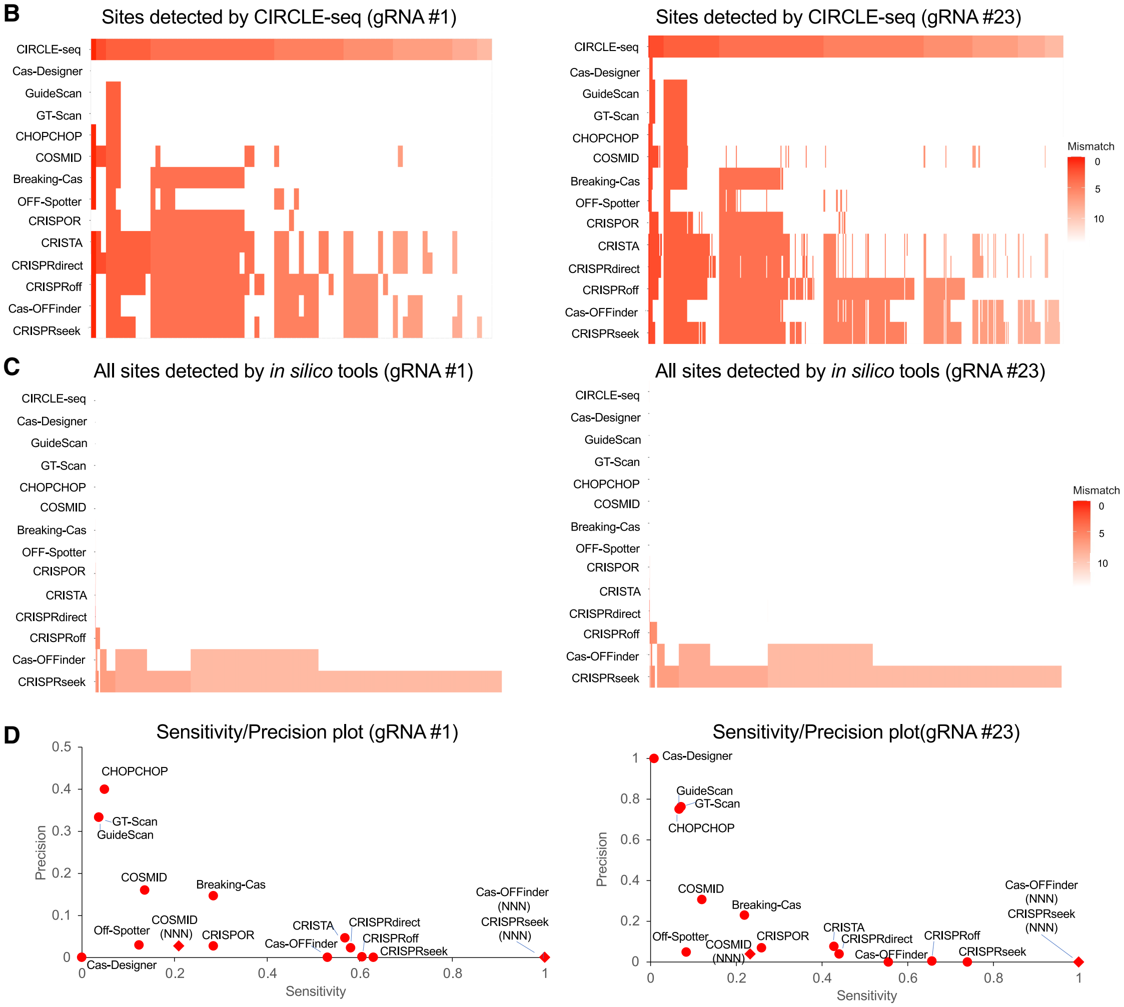

Figure 2. Cross-sectional performance analysis of *in silico* off-target prediction tools

(A) Summary of prediction tools used to identify potential off-target sites in the human genome that are similar to the target sequence of gRNA #1 and #23. For each tool, the default settings of mismatch allowance, PAM specification, and bulge allowance were used. We also utilized CIRCLE-seq analysis to extract the potential DNA cleavage sites in the human genome *in vitro*.

(B) Among the DNA cleavage sites detected by CIRCLE-seq experiments with gRNA #1 (left) and gRNA #23 (right), predicted off-target sites by each tool were displayed as heatmaps. Darker red indicates fewer base pair mismatches. The CIRCLE-seq sites are sorted by the number of mismatches and chromosomal position.

(C) All the predicted genomic sites are plotted for gRNA #1(left) and gRNA #23 (right). Color scale as in (B).

(D) For each prediction tool, precision and sensitivity relative to CIRCLE-seq data were plotted for gRNA #1 (left) and gRNA #23 (right). The analyses performed with the PAM set to NNN are plotted as diamonds.

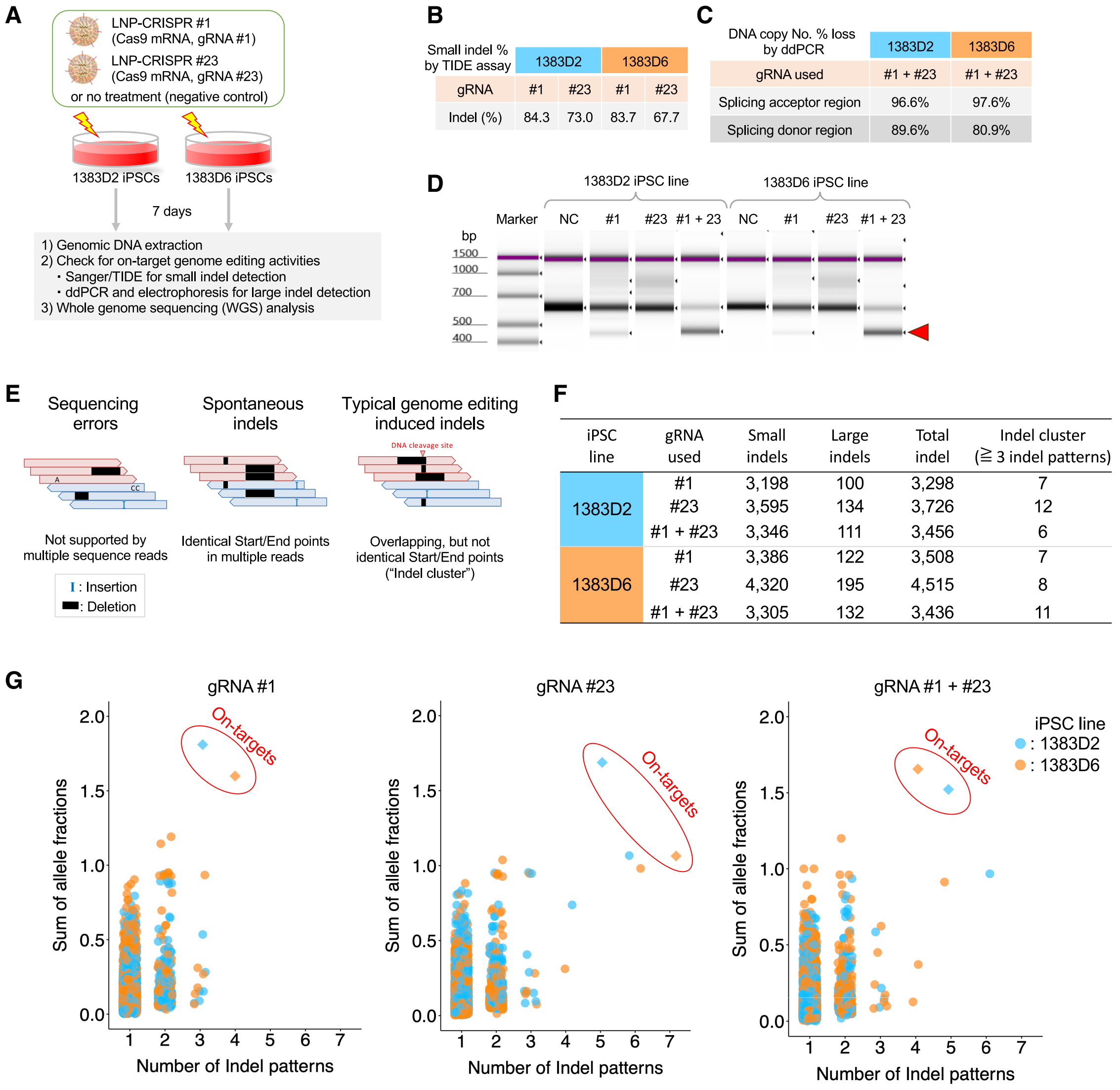

Figure 3. Whole-genome sequencing analysis of human iPSCs treated with LNP-CRISPR

(A) Schematic of the experimental design.

(B) On-target indel frequencies measured by TIDE assay for each gRNA sample.

(C) The genome editing efficiency by the dual gRNA sample was assessed as DNA copy number loss at the exon 45 region by droplet digital PCR (ddPCR) analysis.

(D) TapeStation DNA electrophoresis to assess the DNA cleavage at the dual gRNAs. The arrowhead indicates the DNA band cleaved by gRNA #1 and gRNA #23.

(E) When indels are detected from whole genome sequences, spontaneous indels (i.e., heterozygous indels) are detected as identical indel patterns in multiple sequence reads (middle). Therefore, they can be distinguished from noise, such as randomly occurring sequence errors (left). On the other hand, indels induced by genome editing typically induce different indel patterns across multiple cells. Therefore, overlapping but not identical indel patterns can be detected in one place as an “indel cluster.”

(F) Number of overlapped indels (small, large, or total) obtained by the “*bedtools merge*” function from the WGS analysis. Regions with three or more indel patterns were defined as indel clusters and shown in the far-right column.

(G) A scatterplot of allele fractions versus indel pattern counts for all the indels. The on-target sites are shown as diamonds, and the off-target sites are shown as circles.

distinguish bona fide off-target mutations from spontaneous variants, unedited isogenic controls for each line were sequenced in parallel. We used GATK Mutect2 for detecting small indels (50 bp or less) and Manta for detecting structural variants larger than 50 bp using untreated iPSC lines as controls, to subtract de novo mutations after the LNP-CRISPR treatment (Figure S5).[39,40]

Even when de novo variants are extracted using isogenic controls, sequencing errors and spontaneously acquired mutations during cell culture are inevitably included, making it difficult to determine whether individual variants are truly induced by genome editing. Previous WGS-based studies have generally inferred editing-induced indels based on sequence similarity to the gRNA target site.

However, this approach requires prior knowledge of the gRNA sequence and cannot detect gRNA-independent indels, including those with mismatches beyond the predefined threshold parameters. In this study, we leveraged the observation that genome editing-induced mutations are typically heterogeneous in length and type but concentrated at specific loci, whereas spontaneous mutations are more randomly distributed (Figure 3E). Based on this principle, we developed an "indel cluster method," which identifies multiple adjacent indels as a single cluster site. After variant calling with GATK Mutect2 and Manta, indels of various sizes were merged, yielding approximately 3,000–4,000 sites per condition (Figure 3F). Each site was then characterized by the number of distinct indel patterns and the sum of allele fractions (Figure 3G). Most sites showed only one or two indel patterns, whereas sites with three or more patterns were defined as "indel clusters," reflecting the typical mutational signatures of genome editing. Consistent with this definition, on-target sites exhibited a relatively high number of indel patterns and allele fractions. To validate the method, we examined on-target loci (Figures S4B and S4C): dual gRNA-treated samples displayed multiple large deletions around the two gRNA binding sites, while untreated controls showed no indels. A similar trend was observed in single gRNA-treated samples, albeit with smaller clusters, as expected. In the 1383D2 sample treated with gRNA #23 alone, we detected a large unexpected deletion, which could have been overlooked with amplicon sequencing. Collectively, these results demonstrate that our indel cluster method reliably captures genome-edited positions at on-target sites. Moreover, indel clusters with high allele fractions are considered potential high-risk sites for genome editing-induced off-target events (Figure 3G).

Comprehensive off-target analysis

Finally, we integrated potential off-target sites identified by three complementary approaches: *in silico* prediction using Cas-OFFinder (NNN PAM, up to 9 mismatches), *in vitro* cleavage detected by CIRCLE-seq, and *in cellulo* mutagenesis revealed by WGS indel cluster analysis, corresponding to potential DNA binding, cleavage, and mutagenesis sites, respectively (Figures 4A and S5). Despite maximizing the *in silico* search space, only 11 off-target sites and 2 on-target sites were identified (Figures 4B, 4C, and S6A–S6F). Ten of the 11 off-targets originated from gRNA #23, likely due to reduced sequence complexity from a poly(A) stretch within the spacer. Among these, the lowest mismatch count was two (chr9:11687303–11687325 and chr18:58827057–58827079), while the highest was seven (chr3:67017674–67017696). Consistent with previous reports, mismatches were predominantly located in the non-seed region distal to the PAM. Regarding PAM recognition, 9 of the 11 sites carried the canonical NGG motif, whereas two carried non-canonical PAMs (NGC at chr5:113937846–113937868 and NTG at chr7:140404449–140404471). For exhaustive off-target detection, it is necessary to perform the analysis with the PAM set to NNN rather than restricting it to NGG.

We also confirmed that the cleavage sites in the CIRCLE-seq data are 3–4 bp from the PAM. Using the CIRCLE-seq peak height (read counts) as a quantitative measure of cleavage activity, the on-target sites showed the highest DNA cleavage activity (6,807 for gRNA #1 and 5,152 for gRNA #23), while the highest off-target height was 2,455 at chr1:17729009–17729031.

We initially hypothesized that off-targets detected with each single gRNA would also be recovered under the dual gRNA condition. However, among the 11 off-targets identified with gRNA #23 alone, only 6 were retained in the dual gRNA samples. Apart from the on-targets, only two sites (chr9:11687303–11687325 and chr18:58827057–58827079) were reproducibly detected across all gRNA #23 samples. This may be because the read depth of the WGS analysis is insufficiently high, leading to variability in detection sensitivity across samples.

To address the potential sensitivity limitations of WGS, we performed short-read amplicon sequencing for the on-target and 11 candidate sites (Figures 4D and S7A). Under conditions in which nearly 100% on-target genome editing efficiency was achieved using high-dose LNPs to maximize off-target detection sensitivity, variable indel efficiencies were observed at most of the 11 candidate off-target sites across two iPSC lines. The indel frequencies measured by amplicon sequencing showed low correlation with the cleavage efficiencies observed in CIRCLE-seq (Figure 4C). These discrepancies may reflect differences in epigenetic states. In fact, when we performed the same amplicon sequencing with human myogenic cell line Hu5/KD3 cells, the indel profiles differed from iPSC lines (Figures 4D and S7B–S7D).[41]

Among the detected sites by amplicon sequencing, the locus on chr9 (chr9:11687303–11687325) exhibited a high indel frequency of 58%–85%. This site has been reproducibly detected in WGS analyses of iPSCs, with only two base-pair mismatches in the non-seed region of gRNA #23 (Figure S8A, left). However, another site on chr18 with a two-base-pair mismatch did not show such a high indel rate. Our data highlight the importance of highly sensitive amplicon sequencing analysis and further indicate that off-target mutation patterns can vary across cell types and on-target cleavage activity.

Risk assessment of the detected sites

When annotating the detected off-targets, we found that four of the eleven sites overlapped with a gene or non-coding RNA. Among them, the *GPHN* (gephyrin) gene is listed in the cancer-associated gene database COSMIC, but not in the Shibata cancer-driver gene list.[42,43] The C-terminal domain of *GPHN* (e.g., exon 14–22) has previously been reported to generate fusion transcripts with *MLL* (*KMT2A*), a well-established leukemia-associated driver gene.[44] Notably, the off-target mutation is a single adenine insertion within a poly(A) tract at chr14:67492431–67492453, located within intron 12 of *GPHN*, which was detected only in the 1383D2 iPSC line but not in 1383D6 (Figure S8A, right). Although the displayed insertion site appears slightly shifted from the Cas9 cleavage position, this discrepancy reflects a mapping ambiguity within a homopolymeric tract.

A

Stages of off-target risk in genome editing

1) Binding → *In silico* Off-target tools
2) Cleavage → *In vitro* CIRCLE-seq
3) Mutate → *In cellulo* Genome sequencing
4) Altering gene function
5) Phenotypic manifestation

B

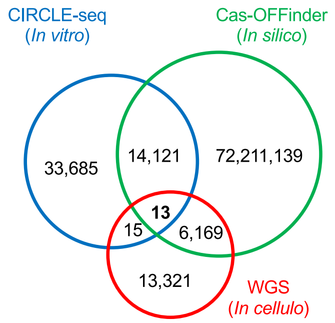

C

		Cas-OFFinder (*In silico*)			CIRCLE-seq (*In vitro*)		WGS (*In cellulo*)						
							iPSC 1383D2			iPSC 1383D6			
gRNA position (hg38)	gRNA	Sequence of the site	Strand	MM	Cleaved site from PAM	Peak height	#1	#23	#1+#23	#1	#23	#1+#23	Overlapping gene
chrX:31968338-31968360	#23	AGCTGTCAGACAGAAAAAAG**AGG**	−	0	3	5152	.	+	+	.	+	+	*DMD* (Exon)
chrX:31968505-31968527	#1	TGGTATCTTACAGGAACTCC**AGG**	−	0	3	6807	+	.	+	+	.	+	*DMD* (Exon)
chr1:17729009-17729031	#1	TGacATCTTACAGGgACTCC**CGG**	+	3	3	2455	.	.	+	.	.	.	.
chr1:84361946-84361968	#23	AcCTGcCAGACAGAAAAAgG**AGG**	+	3	3	1819	.	.	+	.	+	.	.
chr3:67017674-67017696	#23	tagctgCAGgCAGAAAAAAG**AGG**	+	7	3	140	.	+	.	.	.	.	.
chr5:113937846-113937868	#23	AaCTGTCAGAagaAAAAAga**GGC**	+	6	4	758	.	.	+	.	.	.	.
chr6:137734937-137734959	#23	ttacaTCAGACAGAAAAAAG**AGG**	−	5	3	25	.	+	.	.	.	.	*LINC02539* (ncRNA)
chr7:140404449-140404471	#23	AaaaGTCAGACAGgAAAAAa**GTG**	+	5	2	653	.	+	.	.	.	.	*RAB19* (Intron)
chr9:11687303-11687325	#23	AGCTGcCAcACAGAAAAAAG**TGG**	−	2	3	855	.	+	+	.	+	+	.
chr14:67492431-67492453	#23	tcCTGTCAGtCAGAAAAAAG**AGG**	+	3	3	1612	.	+	+	.	.	.	*GPHN* (Intron)*
chr18:58827057-58827079	#23	AtCTGTCAcACAGAAAAAAG**GGG**	−	2	3	1634	.	+	+	.	+	+	*LINC01926* (ncRNA)
chr20:58378315-58378337	#23	tctTGcCAGACAGAAAAAAa**AGG**	−	5	3	1240	.	.	.	.	+	.	.
chrX:96288780-96288802	#23	AGCTacCAGACAGtAAAAAG**GGG**	+	3	3	632	.	+	+	.	.	.	.

D

	iPSC 1383D2				iPSC 1383D6				Hu5	
gRNA position (hg38)	NC	#1	#23	#1+#23	NC	#1	#23	#1+#23	NC	#1+#23
chrX:31968338-31968527 (On-target)	1.01	97.11	99.56	100.00	1.04	99.03	98.90	99.91	1.08	92.73
chr1:17729009-17729031	0.36	8.08	0.40	5.34	0.39	7.41	0.35	12.63	0.34	3.65
chr1:84361946-84361968	0.27	0.27	5.62	5.21	0.32	0.31	11.78	8.26	0.35	3.10
chr3:67017674-67017696	0.58	0.56	0.78	0.96	0.60	0.59	1.82	1.07	0.61	0.69
chr5:113937846-113937868	0.56	0.60	3.46	1.41	0.63	0.53	4.34	3.66	0.57	0.74
chr6:137734937-137734959	0.70	0.55	0.53	0.70	0.57	0.55	0.63	0.56	0.64	0.69
chr7:140404449-140404471	0.70	0.53	3.32	2.32	0.57	0.49	5.75	4.67	0.58	2.19
chr9:11687303-11687325	0.57	0.65	65.67	58.04	0.68	0.66	84.54	81.50	0.65	5.92
chr14:67492431-67492453	0.79	0.65	7.48	4.62	0.66	0.61	14.07	9.96	0.74	1.77
chr18:58827057-58827079	1.37	1.34	8.03	6.38	1.29	1.26	17.80	13.72	1.23	5.47
chr20:58378315-58378337	5.62	5.19	11.75	9.85	5.18	5.17	17.81	13.75	5.49	24.06
chrX:96288780-96288802	0.70	0.69	14.56	10.27	0.86	0.73	28.07	23.20	0.70	3.63

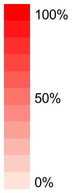

Figure 4. Integrated analysis of off-target sites from the *in silico*, *in vitro*, and *in cellulo* datasets

(A) Genome editing with CRISPR-Cas9 involves DNA binding, cleavage, and mutagenesis, which may alter gene function and, in some cases, lead to phenotypic effects. *In silico* off-target analysis tools typically predict potential binding sites. CIRCLE-seq identifies *in vitro* cleavage sites. WGS detects mutagenesis in cells or tissues.

(B) Venn diagrams of integrated off-target analyses. For CIRCLE-seq and Cas-OFFinder, both gRNA #1 and gRNA #23 are integrated. For the WGS, a total of six datasets, comprising three conditions, gRNA #1 alone, gRNA #23 alone, and gRNA #1 + #23 tested in two iPSC lines (1383D2 and 1383D6), are merged.

(C) Summary of the off-target sites detected by integrated analysis. The two on-target sites are shown in the top two rows highlighted in light beige. MM: the number of mismatched base pairs. Of note, the *GPHN* gene is listed in COSMIC as a fusion partner of *MLL*.

(D) Indel frequencies at the candidate sites identified in Figure 4C were quantified by amplicon sequencing. Values represent the fraction of reads containing indels at each putative Cas9 cutting site. Color intensity reflects indel frequency, with darker red indicating higher values (see color scale on the right). The two on-target sites shown in Figure 4C are presented as a single combined row.

To evaluate whether DNA cleavage and mutagenesis could be induced independently of gRNA sequence similarity, we investigated the integrated dataset from *in silico*, *in vitro*, and *in cellulo* analyses. Specifically, we focused on sites where *in vitro* CIRCLE-seq detected DNA cleavage and *in cellulo* WGS identified mutations, but no homologous sequences were predicted by *in silico* tools. Detailed analyses revealed that, compared with bona fide on-target sites, these loci exhibited lower mapping quality (MapQ), reduced CIRCLE-seq peak heights (read counts), and frequent overlap with repeat elements, problematic genomic regions, or low-mappability

regions (Figure S8B). Thus, the apparently gRNA-independent sites were predominantly false positives, arising from mapping artifacts inherent to massively parallel sequencing. Such repetitive regions remain technically challenging to analyze and require further optimization of the analysis pipeline in future studies.

DISCUSSION

This study establishes a practical, safety-oriented framework for evaluating LNP-delivered dual gRNA CRISPR-Cas9 editing by integrating *in silico* site nomination, *in vitro* genome-wide cleavage profiling, and *in cellulo* whole-genome mutational readouts. Across these layers, we identified a small set of candidate off-target loci and found that most apparent discordances were attributable to mapping and mappability constraints rather than bona fide gRNA-independent editing. Collectively, our results support the translational potential of LNP delivery for exon-skipping strategies while underscoring the need for multi-modal verification of off-target risk.

A key observation is the favorable on-target safety profile of LNP relative to the AAV vector. LNP supported repeat dosing without the marked reduction in indel frequency seen after a second AAV administration, consistent with lower immunogenicity.[21]

In addition, we found that LNP resulted in a lower proportion of insertion events than AAV. Although it was not possible to achieve strict molar normalization between LNP and AAV doses, analysis of indel profiles across multiple AAV doses revealed that even when overall indel frequency was similar to that observed with LNP, the proportion of insertion events was approximately 7-fold higher with AAV (1×10^9 v.g.; Figure S1A). Moreover, analysis of the inserted sequences from AAV again revealed an enrichment for vector-derived sequences mapping to the ITR—particularly the Rep-binding elements—implicating these structured regions as a recurrent source of insertional by-products.[7–9,23] While the mechanistic basis of vector-fragment capture requires further study, these findings highlight a delivery-platform effect on the qualitative spectrum of DNA repair outcomes, not merely on editing efficiency. Beyond genome editing efficiency, LNP and AAV differ in their kinetics and tissue tropism. In terms of kinetics, Cas9 expression following LNP delivery is transient because Cas9 is supplied as mRNA. By contrast, AAV-delivered single-stranded DNA can persist long term as stable episomal concatemers in the nucleus. Importantly, LNPs have been reported to exhibit high accumulation in the mouse liver. Therefore, even with intramuscular administration, careful attention must be paid to the potential for genome editing in non-target tissues, such as the liver, in addition to skeletal muscle. In this study, we utilized the AAV-DJ chimeric serotype to deliver CRISPR-Cas9, but its tropism differs from clinically approved serotypes such as AAV9 and AAVrh74. Considering tissue distribution and transduction efficiency vary by AAV serotype, it is important to use the corresponding serotype when employing AAV vectors as a candidate drug.

We also document dual gRNA-driven structural outcomes, including inversions spanning the region between the two gRNA cut sites, which agree with previous dual gRNA approaches.[8,45–48] For applications in DMD, such inversion events can still contribute to exon-skipping and frame restoration, thereby broadening the range of productive editing outputs. As previously reported in mouse zygotes and human cell lines (HEK293T, HeLa, and U2OS), we observed low-frequency insertion of mouse transposons. The impact of such insertion events on exon skipping should be carefully investigated.[49,50]

Benchmarking *in silico* predictors against CIRCLE-seq revealed the expected trade-off between sensitivity and precision, similar to a previous study.[25] There is a generally weak, guide-dependent correlation between tool scores and quantitative cleavage activity, as reported.[51] From a clinical safety perspective, missing true off-targets is the greater hazard than false positives; we therefore prioritized a high-sensitivity genome-wide search (e.g., Cas-OFFinder under permissive PAM/mismatch settings) and compensated for the low precision by layering *in vitro* and *in cellulo* evidence. This aligns with regulatory expectations that computational nomination be complemented by at least one unbiased experimental assay.[18,19]

WGS analysis of subclones or offspring offers high sensitivity for detecting rare off-target variants because individual alleles are clonally expanded and sequenced, but this approach is limited by sampling bias of subclones and does not capture the diversity of mutational outcomes present in bulk populations.[9,52–57] In contrast, the sensitivity of bulk WGS is dependent on sequencing depth and the overall on-target editing efficiency. When on-target editing efficiency is low, bona fide off-target mutations may remain below the detection threshold in bulk WGS.

To improve the interpretability of WGS in bulk-edited cells, we introduced an indel-cluster method that collapses multiple adjacent indels into a single locus-level signal and quantifies both the diversity of indel patterns and their cumulative allele fraction. On-target sites showed the highest cluster complexity and burden, supporting the utility of this signature for nominating editing-related events. Importantly, performing WGS in isogenic, karyotypically normal iPSCs avoids confounders common to immortalized cancer lines (aberrant repair pathways, chromosomal abnormalities) and better approximates physiological DNA repair.[57–59]

An important finding is that putative gRNA-independent sites—defined as loci with *in vitro* cleavage and *in cellulo* mutations but no *in silico* sequence similarity—were enriched in low-mappability, repetitive, or otherwise problematic regions, had low mapping quality and modest CIRCLE-seq signal, and frequently lay within homopolymers. These features are canonical risk factors for alignment artifacts in massively parallel sequencing. Thus, most such loci likely represent false positives rather than true gRNA-independent mutagenesis. This emphasizes that genome-wide assays must be interpreted through the lens of mappability and alignment uncertainty, especially in repetitive sequence regions, including transposons.

Our risk assessment further illustrates how integrated evidence should temper gene-level concern. Four of eleven off-target candidates overlapped genes or ncRNAs. One, within intron 12 of *GPHN*, drew attention because *GPHN* is listed in COSMIC, and rare *MLL-GPHN* fusions have been reported in two cases.[60,61] However, the observed change of a single adenine insertion within a poly(A) tract was detected in only one iPSC line. We found no accompanying events in *MLL*. On balance, the clinical relevance of this site appears limited, though it remains reasonable to include it in any targeted follow-up panel.

This work still has several technical limitations. First, bulk WGS—even at 60–80×—has finite sensitivity for low-frequency variants, and alignment uncertainties constrain confident calling in repetitive or low-complexity regions. For greater sensitivity and accuracy, amplicon sequencing and long-read sequencing could be implemented. Second, off-target landscapes can be guide- and context-dependent, as reflected by differences in indel efficiencies between iPSCs and Hu5 myogenic cells. Because *in vivo* tissues comprise a wide variety of cell types, determining which cultured cell type to use to assess off-target mutation risk remains a challenge. Broader generalization will require testing additional cell types with multiple gRNAs. Third, assessing the clinical relevance of detected off-target events remains difficult. For example, although rare *MLL-GPHN* fusions have been reported, determining whether such mutations pose a tangible risk to patients remains challenging, complicating go/no-go decisions in clinical development.

For *ex vivo* genome editing therapies, the final cell product can be directly analyzed for off-target safety, but *in vivo* therapies pose greater challenges since key tissues such as skeletal muscle are not readily accessible for repeated biopsies.[62] Although animal models are valuable for general safety assessment, species-specific genomic differences limit direct evaluation of human off-target risks. Thus, precise preclinical assessments in human cells are essential. Because no single method reliably predicts bona fide off-target sites, a multilayered approach is critical, as reflected in recent clinical trials.[23,63,64]

In conclusion, by combining high-sensitivity *in silico* nomination, unbiased *in vitro* cleavage mapping, isogenic iPSC WGS with indel-cluster features, and targeted amplicon sequencing, we present a conservative, scalable path to off-target risk assessment for LNP-delivered CRISPR therapies. We anticipate that this workflow will aid the standardization of preclinical safety packages for *in vivo* genome editing and inform rational progression toward clinical translation of exon-skipping strategies in DMD and beyond.

METHODS

LNP formulation

Two gRNAs targeting human *DMD* exon 45 (#1, #23) and SpCas9 mRNA were encapsulated into lipid nanoparticles as described previously.[21] Briefly, ionizable lipid (60%), DPPC (10.6%), cholesterol (28.7%), and DMG-PEG (0.7%) were dissolved in 90% ethanol. gRNAs and SpCas9 mRNA were dissolved in 10 mM MES buffer (pH 5.5). These organic lipids and aqueous RNAs were mixed using NanoAssemblr Benchtop (Precision Nanosystems, Vancouver, Canada).

Construction and packaging of AAV vectors

The gRNAs (#1 and #23) were synthesized by Eurofins Genomics (Tokyo, Japan) and cloned into the pAAV-Guide-it-Down vector (Takara Bio) using the AAVpro CRISPR-Cas9 system. pAAV-Guide-it-Up and pAAV-Guide-it-Down plasmids were obtained from Takara Bio. Recombinant AAV vectors were packaged into the AAV-DJ serotype (Cell Biolabs) using AAVpro293 cells (Takara Bio) and purified with the AAVpro Purification Kit (Takara Bio).

Approval of animal experiments

All animal experiments conformed to the Association for Assessment and Accreditation of Laboratory Animal Care guidelines and were approved by the Institutional Animal Care and Use Committee in Takeda Pharmaceutical Company, Ltd (Kanagawa, Japan).

In vivo genome editing and extraction of genomic DNA

CAG-Luc2-hDMD Ex45 KI reporter mice were generated by targeting the *Gt(ROSA)26Sor* locus with pCAGGS-Luc2-hEx45 reporter construct as described previously.[20] For *in vivo* genome editing, 10 μg of LNP or 1 × 10 ^11 vector genomes (vg) of AAV were injected into the left gastrocnemius (GC) muscle of 6-week-old male CAG-Luc2-hDMD Ex45 knock-in mice under 2.5% isoflurane anesthesia (four independent mice per group). Four weeks later, the same amount of LNP or AAV was injected into the contralateral (right) GC muscle of the same mice. A total of 4 mice ($n = 4$) were treated simultaneously. Genomic DNA was isolated from the left and right injected muscles using the QIAamp Fast DNA Tissue Kit (QIAGEN).

Preparation of the amplicon-seq library for on-target indel pattern analysis

Amplicon libraries from the 4 mice (left and right legs separately) were prepared by nested PCRs. For the first PCR, the on-target region was amplified in a 50 μL reaction containing 50 ng of genomic DNA, 0.2–0.3 μM of primers (forward: 5′-CTCTTTCCCTACACGACGCTCTTCCGATCTNNNNAATAAAAAGACATGGGGCTTCA-3′; reverse: 5′-CTGGAGTTCAGACGTGTGCTCTTCCGATCTNNNNCCTTTCACCCTGCTTATAATCTC-3′; where NNNN denotes 4-bp indices for sample barcoding), and 1 μL of PrimeSTAR GXL DNA polymerase (TaKaRa, Japan). PCR cycling conditions were: initial denaturation at 94°C for 2 min, followed by 25 cycles of 98°C for 10 s, 60°C for 15 s, and 68°C for 30 s. PCR products were purified using the Wizard SV Gel and PCR Clean-Up System (Promega) without gel extraction.

For the second PCR, Illumina adaptor sequences were attached in a 50 μL reaction containing 50 ng of purified first-round PCR product, primers (forward: 5′-AATGATACGGCGACCACCGAGATCTACANNNNNNNNCTCTTTCCCTACACGACGCTC-3′; reverse: 5′-CAA

GCAGAAGACGGCATACGAGATGTGANNNNNNNNCTGGAGTTCAGACGTGTGCTC-3′; where NNNNNNNN represent 8-bp indices), and 1 μL of PrimeSTAR GXL DNA polymerase. Cycling conditions: initial denaturation at 94°C for 2 min, followed by 25 cycles of 98°C for 10 s, 60°C for 15 s, and 68°C for 30 s.

The resulting libraries were quantified using the KAPA Library Quantification Kit for Illumina (KK4835) and pooled. Sequencing was performed on an Illumina MiSeq platform using the MiSeq Reagent Kit v3 (600-cycle, paired-end) with a 30% PhiX spike-in. FASTQ files were generated with standard Illumina pipelines.

On-target amplicon-seq analysis

Low-quality reads in the FASTQ files were removed using cutadapt (v2.4) with options -q 30 -m 20. Demultiplexing by barcode was performed with fastx_barcode_splitter.pl (FASTX-Toolkit; options: –mismatches 0 –partial 0). Paired-end reads were collected using fastq_pair (v0.20), merged with Flash2 (v2.2.00), and barcode sequences were trimmed with custom Perl/Python scripts. CRISPResso (v1.0.13) was run with the human *DMD* exon 45 sequence as the reference. Reads were categorized into five groups (unmodified, mutated_only, deletion_only, indel_both, insertion_only) based on "Alleles_frequency_table.txt," and the average proportions across replicates (n = 4) were calculated. For insertion events >10 bp, sequences were extracted from "Alleles_frequency_table.txt" and analyzed with BLASTn (v2.9.0).

1) Against a custom database of human *DMD* exon 45 and AAV full-Cas9 sequences using options -task blastn-short -outfmt 6 -evalue 0.01 -max_target_seq 1.
2) Against the mouse reference genome (GRCm39.primary_assembly.genome.fa, GENCODE) with options -evalue 0.01 -max_target_seqs 10 -reward 2 -penalty −3 -gapopen 2 -gapextend 4 -word_size 4 -dust no -soft_masking false.
3) Annotation with "gencode.vM37.primary_assembly.annotation.gff3" (GENCODE) and rmsk.txt (UCSC RepeatMasker).
4) For sequences passing both steps, BLASTn was run against gencode.vM37.transcripts.fa (GENCODE) under the same conditions.

Sequences not aligned with >80% coverage in the above searches were classified as "unknown." The average proportions of each origin were calculated across replicates (n = 4). To identify insertion positions within the AAV vector, inserted sequences were aligned with ClustalW2 (v2.1) and analyzed with BLASTn as described. Results were converted into bedGraph files, merged across replicates (n = 4), and visualized as histograms of insertion size and positional frequency maps using R (v3.6.1).

In the AAV 1×10^{10} vg group, one mouse died during the experiment; therefore, the sample size was n = 3.

CIRCLE-seq data analysis

CIRCLE-seq was performed essentially as described previously, with two additional processes implemented in this study.[21] First, Cas-OFFinder (v2.4) was used to obtain PAM positions and the number of mismatches between gRNA sequences and candidate binding sites. Multi-FASTA files generated from CIRCLE-seq data were used as input, with PAM set to "NNN" and mismatch allowance to 9. Second, we calculated the distance between each predicted PAM and the putative Cas9 cleavage site, defined by the 5′ ends of CIRCLE-seq reads. Specifically, left-side peaks were extracted from BAM files with FLAG values "83" or "147." Peaks were retained when the distance between the PAM and cleavage site was ≤6 bp, consistent with known Cas9 cleavage positions. The resulting CIRCLE-seq peaks, processed with these steps, were used as the set of experimentally detected off-target sites.

In silico prediction of off-target sites

Off-target prediction tools were collected using WeReview database. The following tools were applied with the specified parameters:

- Cas-OFFinder (v2.4): genome = hg38.fa, PAM = NGG, mismatches ≤9
- CHOPCHOP (v3): target = human *DMD* exon 45, genome = hg38/GRCh38, CRISPR-Cas9 knockout mode, mismatches ≤3
- GT-Scan (Web-based tool, accessed in October 2020): species = human, chrX:31968300–31968600 (*DMD* exon 45), ruleset = SpCas9 (NGG), off-target filter = NRG, mismatch limit = 3
- CRISPRdirect (Web-based tool, accessed in September 2020): input sequence = ±100 bp around *DMD* exon 45, PAM = NGG, genome = hg38, search both strands, mismatches/gaps ≤4, target length = 20 bp + PAM
- Off-Spotter (Web-based tool, accessed in October 2020): genome = hg38, PAM = NGG, mismatches ≤5
- Cas-Designer (Web-based tool, accessed in October 2020): nuclease = SpCas9 (5′-NGG-3′), genome = hg38, target = same as GT-Scan region, allowing 1 nt bulge
- GuideScan (Web-based tool, accessed in October 2020): coordinates = chrX:31968300–31968600, genome = hg38, enzyme = SpCas9
- COSMID (Web-based tool, accessed in September 2025): genome = hg38, PAM = NGG, allowed mismatches: no indels = 3, 1-base deletion = 2, 1-base insertion = 2
- Breaking-Cas (Web-based tool, accessed in October 2020): genome = human (9606), nuclease = SpCas9 (NGG, PAM at 3′), guide length = 20, mismatches ≤4
- CRISPOR (v4.98): installed locally (conda, python 2.7); genome = hg38; input = ± 100 bp FASTA around *DMD* exon 45; mismatches ≤6; off-targets extracted using guideID for gRNA #1 and #23
- CRISTA (Web-based tool, accessed in October 2020): input = gRNA #1 and #23, guide length = 20 nt, genome = hg38
- CRISPRoff (v1.1): input = gRNA #1 and #23, genome = hg38
- CRISPRseek (v1.26.0 for the default PAM and v1.44.0 for NNN PAM): run in R Bioconductor 3.10 or 3.19 with offTargetAnalysis using parameters: input gRNA FASTA, findgRNAsWithREcutOnly = FALSE, findPairedgRNAOnly = FALSE, genome = hg38 (BSgenome.Hsapiens), transcript annotation = TxDb.Hsapiens.UCSC.hg38.knownGene, topN set high to capture all potential sites

Sensitivity and precision plot of each tool

Sensitivity and precision were evaluated by comparing CIRCLE-seq-detected cleavage sites (treated as true positives, TP) with predicted sites from *in silico* tools. For each tool, the numbers of false positives (FP) and false negatives (FN) were also recorded. Precision was calculated as TP/(TP + FP), and sensitivity as TP/(TP + FN).

Correlation between predicted scores and CIRCLE-seq data

The correlation between prediction scores and experimental cleavage activity was evaluated by comparing tool-derived scores with CIRCLE-seq peak heights. Prediction scores from CRISTA, CRISPRseek, CRISPRoff, CRISPOR (MIT and CFD scores), COSMID, and Breaking-Cas were used. In addition, uCRISPR was applied to estimate gRNA-target affinity for sites detected in the CIRCLE-seq analysis. Off-target sites detected by each tool were plotted using R (v3.6.1), and correlation coefficients were calculated with the R function "cor.test()" with option method = "pearson."

Cell culture and CRISPR-Cas9-mediated genome editing

Human iPSCs (clones 1383D2 and 1383D6) were cultured in StemFit AK02N medium (Ajinomoto) supplemented with 10 μM Y-27632 (Fujifilm Wako) on iMatrix511-coated plates (Nippi). Cells were seeded at 5×10^4 cells per well and cultured overnight. Just before LNP addition, the medium was replaced with fresh medium containing 15 μg of total RNA (gRNA #1 + Cas9 mRNA or gRNA #23 + Cas9 mRNA) and incubated overnight. The following day, the medium containing LNPs was removed, and cells were harvested 7 days post-treatment. Genomic DNA was extracted using the NucleoSpin Tissue kit (Takara Bio).

Evaluation of genome editing

Cleavage activity of gRNA #1 and gRNA #23 individually was evaluated by TIDE (Tracking of Indels by Decomposition). The target regions were PCR amplified from purified genomic DNA using primers (forward: 5′-GTTAGTGCCTTTCACCCTGC-3′; reverse: 5′-CCGCAGATTCAGGCTTCCC-3′), followed by Sanger sequencing and TIDE analysis.

For dual gRNA editing (gRNA #1 and #23), cleavage activity was quantified by droplet digital PCR (ddPCR, Bio-Rad QX200). Genomic DNA was amplified using primers and probes specific for non-target sites (forward: 5′-GACATGCCCATATATCCAAAGGA-3′; reverse: 5′-AACCGAGAGGGTGCTTTTTTC-3′; VIC probe: 5′-ACAAGAC AGAAAGACACCTT-3′), gRNA #1 sites (forward: 5′-TTTGCCGC TGCCCAAT-3′; reverse: 5′-CATTTTTGTTTTGCCTTTTTGGT-3′; FAM probe: 5′-CCATCCTGGAGTTCC-3′), and gRNA #23 sites (forward: 5′-AAAATTGGGAAGCCTGAATCTG-3′; reverse: 5′-TTA GATCTGTCGCCCTACCTCTTT-3′; FAM probe: 5′-AGGTCTGC AAACAGCT-3′).

Evaluation of cytotoxicity and pluripotent marker in LNP-treated iPSCs

Human iPS cell lines (1383D2 and 1383D6) were seeded at 1×10^5 cells per well in 24 well plates coated with iMatrix-511 (Nippi) and cultured overnight in StemFit AK02N medium (Ajinomoto) supplemented with 10 μM Y-27632 (Fujifilm WAKO). The next day, after changing to fresh medium, LNPs encapsulating either 7.5 or 15 μg of total RNA (Cas9 mRNA + sgRNA #1 + sgRNA #23 or Cas9 mRNA alone) were added to each well. After overnight incubation, the LNP-containing medium was removed, and cell counting was performed. To evaluate the expression of a pluripotency marker, cells were harvested 4 days after LNP treatment, stained with anti-TRA-1-60 antibody (1:20 dilution, BD, #560380) for 20 min, and analyzed by flow cytometry.

Library preparation and mapping

WGS libraries were prepared by Macrogen Japan Corp. using the Illumina TruSeq DNA PCR-Free kit (350 bp insert). Genomic DNA was fragmented with a Covaris system, end repaired to blunt ends, and size selected with purification beads. An "A" base was added to the 3′ ends, followed by ligation of indexing adapters with a complementary "T" overhang. Adapter-ligated fragments were loaded onto flow cells and sequenced on an Illumina HiSeq platform. Base calls (BCL/cBCL files) were converted to FASTQ format using bcl2fastq2 (v2.20.0) with the demultiplexing parameter –barcode-mismatches 0 (perfect match). Paired-end FASTQ files were mapped to the human genome (hg38; UCSC assembly, based on GRCh38, Dec 2013) using the iSAAC aligner (v04.18.11.09). Final alignment files were generated in BAM format.

Variant calling for small indels

Small indels were detected using GATK Mutect2 (gatk4-4.2.5.0, MutectVersion = 2.2). BAM files were first processed with Base Quality Score Recalibration (BQSR) using gatk BaseRecalibrator and gatk ApplyBQSR. For recalibration, the following reference files were provided to the –known-sites option: Homo_sapiens_assembly38.dbsnp138.vcf, Mills_and_1000G_gold_standard.indels.hg38.vcf, and Homo_sapiens_assembly38.known_indels.vcf, all downloaded from Broad Genomics hg38 resources. To accelerate variant calling, BQSR-treated BAM files were split by chromosome using samtools (v1.14). Variant calling was then performed with gatk Mutect2 using the option –tumor-lod-to-emit 3, followed by filtering with gatk FilterMutectCalls. The resulting per-chromosome VCF files were merged into a genome-wide VCF using vcf-merge.

VARIANT CALLING FOR STRUCTURAL VARIANTS

Structural variants (SVs) were detected with Manta (v1.6.0). Briefly, workflows were initialized with configManta.py (options: –normalBam, –tumorBam, –referenceFasta, –runDir) and executed with runWorkflow.py. The resulting somaticSV.vcf.gz was used for downstream SV analysis.

Indel cluster preparation

Indel clusters were generated by converting VCF files (from Mutect2 and Manta) into bedGraph format using a custom Python script based on cyvcf2 (v0.30.14).

For Mutect2 VCFs, allele fraction (AF) values were extracted, summed across variants, and variants labeled as normal_artifact were excluded. For Manta VCFs, paired-read (PR) and split-read (SR) support values were extracted for both ALT and REF alleles. Then, the indel allele fraction was calculated by dividing the sum of PR and SR of ALT by that of ALT and REF. The number of distinct indel patterns was also counted for both tools. Finally, Mutect2 and Manta indel clusters were merged using bedtools (v2.27.1) and analyzed as *in cellulo* WGS data.

Detecting off-target sites with the comprehensive analysis

To perform comprehensive off-target analysis, CIRCLE-seq data were reprocessed from FASTQ files obtained in our previous study.[21] Reads with mapping quality (MapQ) = 0 were discarded. CIRCLE-seq peaks were then generated as bedGraph files using a custom Python script, which extracted the 5′ positions of R1 and R2 reads. Peaks supported by fewer than 10 reads in total across two replicates were excluded. Cas-OFFinder (NNN PAM, up to 9 mismatches) predictions for gRNA #1 and gRNA #23 were also converted into bedGraph files using custom scripts. Comprehensive off-target sites were defined as genomic loci overlapping among WGS indel clusters, CIRCLE-seq peaks, and Cas-OFFinder predictions using bedtools intersect (Figures S5 and S6). Because CIRCLE-seq and WGS peaks are often narrow, they can be misclassified as non-overlapping despite residing within the same Cas-OFFinder-predicted site. To address this, 20 bp margins were added upstream and downstream of each CIRCLE-seq peak prior to intersection. These margins were removed after bedtools intersect was completed.

Preparation of genomic DNA from Hu5 cells treated with LNP-CRISPR-Cas9

Hu5/KD3 cells were cultured in D-MEM (high glucose; FUJIFILM Wako) supplemented with 20% fetal bovine serum (FBS) and 2% Ultroser G (Sartorius, 15950-017) in collagen-coated 100 mm dishes (Corning BioCoat, 356450). For LNP treatment, Hu5 cells were seeded in collagen-coated 6-well plates at a density of 3×10^5 cells per well and cultured overnight to allow cell attachment. The culture medium was then replaced with fresh D-MEM (high glucose) containing 10% FBS, and LNP formulations were added to the medium at a final RNA concentration of 100 ng/μL. Three LNP treatment conditions were used: gRNA #1 + Cas9 mRNA, gRNA #23 + Cas9 mRNA, and gRNA #1 + gRNA #23 + Cas9 mRNA. For the dual-gRNA condition, the two gRNAs were mixed at equal proportions.

After overnight incubation with LNPs, the culture medium was replaced with fresh medium, and cells were further cultured for 2 days. Cells were passaged into collagen-coated 100 mm dishes at a density of 2.5×10^5 cells per dish to expand cell numbers. After an additional 3 days of culture, cells were harvested. To achieve sufficient on-target genome editing efficiency, the LNP treatment was repeated twice. After the second round of LNP treatment, cells were harvested, and genomic DNA was extracted using the NucleoSpin Tissue kit (Macherey-Nagel, 740952.50).

Amplicon sequencing of on- and off-target sites identified by the integrated analysis

Genomic regions encompassing the on-target and candidate off-target sites identified by genome-wide analysis were amplified using locus-specific primers (Figure S7A). Genomic DNA from template iPSCs was identical to that used for WGS. Genomic DNA from Hu5 cells was prepared as described above.

Amplicon library preparation was performed by FASMAC Co., Ltd. using a two-step PCR protocol with ExTaq HS DNA Polymerase (Takara Bio) and TruSeq DNA HT Sample Prep Kit (Illumina). In the first PCR, genomic DNA (5 μL; 1–5 ng/μL) template and primers (forward: 5′- ACACTCTTTCCCTACACGACGCTCTTCC GATCT-[target]-3′ and reverse: 5′- GTGACTGGAGTTCAGACGT GTGCTCTTCCGATCT-[target]-3’; where [target] sequences are provided in Figure S7A) were used in a 20 μL reaction. The PCR conditions were 94°C for 2 min; 25 cycles of 94°C for 30 s, annealing at 60°C–63°C for 30 s, and 72°C for 30 s; followed by 72°C for 5 min. PCR products were purified using magnetic beads and used as templates for the second PCR to add sequencing adapters by using the second PCR primers (forward: 5′- AATGATACGGCG ACCACCGAGATCTACACNNNNNNNNACACTCTTTCCCTAC ACGACGC-3′, and reverse: 5′- CAAGCAGAAGACGGCATACGA GATNNNNNNNNGTGACTGGAGTTCAGACGTGTG-3’; where NNNNNNNN represent 8-bp indices). The second PCR was performed under the following conditions: 94°C for 2 min; 8 cycles of 94°C for 30 s, 60°C for 30 s, and 72°C for 30 s; followed by 72°C for 1 min. Libraries were sequenced on an Illumina MiSeq i100 Plus platform with paired-end 2 × 300 bp reads. Adapter trimming was performed by FASMAC, and FASTQ files were provided for downstream analysis.

CRISPResso2 (version 2.3.3) was used to analyze amplicon sequencing data. Approximately average 7.7×10^4 reads per library for iPSC and 12.6×10^4 reads per library for Hu5 were used in CRISPResso2 analyses. All insertions and deletions overlapping a 40 bp window (20 bp upstream and 20 bp downstream) surrounding the target site, starting 3–4 bp from the PAM, were collected. The proportions of reads containing indels relative to the total number of reads were calculated for each library.

Checking problematic regions and mappability

Problematic regions and mappability were evaluated using the UCSC Genome Browser (https://genome.ucsc.edu/). Regions annotated as “LowMap+SegDup” or “All difficult regions” were classified as problematic. Mappability scores were obtained either by direct inspection in the browser or by averaging values from bigWig tracks downloaded from the same resource. Repetitive sequence annotations were retrieved from UCSC RepeatMasker (rmsk).

Statistical analysis

Statistical analyses were performed in R (ver. 3 or 4) using the multcomp package. Data are presented as mean ± SD to show the degree of variation. One-way ANOVA followed by Dunnett’s multiple

comparisons was used to compare each treatment group with the PBS-treated control group in Figure S3. $p < 0.05$ was considered statistically significant.

DATA AND CODE AVAILABILITY

The NGS data used in this study have been deposited in the DDBJ BioProject database (DDBJ BioProject: PRJDB35922).

ACKNOWLEDGMENTS

We would like to thank Shinya Yamanaka and Yasushi Kajii for their generous support and fruitful discussion. This study was supported by the T-CiRA Joint Research Program (to A.H.) by Takeda Pharmaceutical Company and in part by AMED under grant number JP19im0210115 (to A.H. and N.I.), JP24am0521006 (to A.H.), JP22bm1123006 (to A.H.), and JP23bm1323001 (to A.H.).

AUTHOR CONTRIBUTIONS

Y.N. performed bioinformatic analysis. N.F. performed the iPSC experiments and WGS library preparation. Y.M. performed the mouse experiments. D.L. performed the Hu5 experiments. N.I. and A.H. conceived the project and supervised the team. Y.N. drafted and A.H. edited the manuscript.

DECLARATION OF INTERESTS

The authors declare the following competing interests: N.I. is an employee of Takeda Pharmaceutical Company. Y.M. was an employee of Takeda Pharma during the project and is now an employee of Axcelead Inc. N.F., Y.M., and A.H. have filed patent applications regarding the formulation of LNP. The other authors declare no competing interests.

DECLARATION OF GENERATIVE AI AND AI-ASSISTED TECHNOLOGIES IN THE WRITING PROCESS

During the preparation of this work, the authors used ChatGPT, DeepL, and Grammarly in order to translate, check the contexts, and polish the writing. After using these tools, the authors reviewed and edited the content as needed and take full responsibility for the content of the publication.

SUPPLEMENTAL INFORMATION

Supplemental information can be found online at https://doi.org/10.1016/j.omtn.2026.102958.

REFERENCES

1. Jinek, M., Chylinski, K., Fonfara, I., Hauer, M., Doudna, J.A., and Charpentier, E. (2012). A programmable dual-RNA–guided DNA endonuclease in adaptive bacterial immunity. Science *337*, 816–821. https://doi.org/10.1126/science.1225829.

2. Ousterout, D.G., Kabadi, A.M., Thakore, P.I., Majoros, W.H., Reddy, T.E., and Gersbach, C.A. (2015). Multiplex CRISPR/Cas9-based genome editing for correction of dystrophin mutations that cause Duchenne muscular dystrophy. Nat. Commun. *6*, 6244. https://doi.org/10.1038/ncomms7244.

3. Nelson, C.E., Hakim, C.H., Ousterout, D.G., Thakore, P.I., Moreb, E.A., Castellanos Rivera, R.M., Madhavan, S., Pan, X., Ran, F.A., Yan, W.X., et al. (2016). In vivo genome editing improves muscle function in a mouse model of Duchenne muscular dystrophy. Science *351*, 403–407. https://doi.org/10.1126/science.aad5143.

4. Tabebordbar, M., Zhu, K., Cheng, J.K.W., Chew, W.L., Widrick, J.J., Yan, W.X., Maesner, C., Wu, E.Y., Xiao, R., Ran, F.A., et al. (2016). In vivo gene editing in dystrophic mouse muscle and muscle stem cells. Science *351*, 407–411. https://doi.org/10.1126/science.aad5177.

5. Rodrigues, M., and Yokota, T. (2018). An overview of recent advances and clinical applications of exon skipping and splice modulation for muscular dystrophy and various genetic diseases. In Exon Skipping and Inclusion Therapies Methods in Molecular Biology, T. Yokota and R. Maruyama, eds. (Springer New York), pp. 31–55. https://doi.org/10.1007/978-1-4939-8651-4_2.

6. Long, C., Amoasii, L., Mireault, A.A., McAnally, J.R., Li, H., Sanchez-Ortiz, E., Bhattacharyya, S., Shelton, J.M., Bassel-Duby, R., and Olson, E.N. (2016). Postnatal genome editing partially restores dystrophin expression in a mouse model of muscular dystrophy. Science *351*, 400–403. https://doi.org/10.1126/science.aad5725.

7. Hanlon, K.S., Kleinstiver, B.P., Garcia, S.P., Zaborowski, M.P., Volak, A., Spirig, S.E., Muller, A., Sousa, A.A., Tsai, S.Q., Bengtsson, N.E., et al. (2019). High levels of AAV vector integration into CRISPR-induced DNA breaks. Nat. Commun. *10*, 4439. https://doi.org/10.1038/s41467-019-12449-2.

8. Nelson, C.E., Wu, Y., Gemberling, M.P., Oliver, M.L., Waller, M.A., Bohning, J.D., Robinson-Hamm, J.N., Bulaklak, K., Castellanos Rivera, R.M., Collier, J.H., et al. (2019). Long-term evaluation of AAV-CRISPR genome editing for Duchenne muscular dystrophy. Nat. Med. *25*, 427–432. https://doi.org/10.1038/s41591-019-0344-3.

9. Singh, K., Fronza, R., Evens, H., Chuah, M.K., and VandenDriessche, T. (2024). Comprehensive analysis of off-target and on-target effects resulting from liver-directed CRISPR-Cas9-mediated gene targeting with AAV vectors. Mol. Ther. Methods Clin. Dev. *32*, 101365. https://doi.org/10.1016/j.omtm.2024.101365.

10. Hsu, P.D., Scott, D.A., Weinstein, J.A., Ran, F.A., Konermann, S., Agarwala, V., Li, Y., Fine, E.J., Wu, X., Shalem, O., et al. (2013). DNA targeting specificity of RNA-guided Cas9 nucleases. Nat. Biotechnol. *31*, 827–832. https://doi.org/10.1038/nbt.2647.

11. Fu, Y., Foden, J.A., Khayter, C., Maeder, M.L., Reyon, D., Joung, J.K., and Sander, J.D. (2013). High-frequency off-target mutagenesis induced by CRISPR-Cas nucleases in human cells. Nat. Biotechnol. *31*, 822–826. https://doi.org/10.1038/nbt.2623.

12. Torres-Perez, R., Garcia-Martin, J.A., Montoliu, L., Oliveros, J.C., and Pazos, F. (2019). WeReview: CRISPR tools—Live repository of computational tools for assisting CRISPR/Cas experiments. Bioengineering *6*, 63. https://doi.org/10.3390/bioengineering6030063.

13. Tsai, S.Q., Nguyen, N.T., Malagon-Lopez, J., Topkar, V.V., Aryee, M.J., and Joung, J.K. (2017). CIRCLE-seq: a highly sensitive in vitro screen for genome-wide CRISPR–Cas9 nuclease off-targets. Nat. Methods *14*, 607–614. https://doi.org/10.1038/nmeth.4278.

14. Kim, D., Bae, S., Park, J., Kim, E., Kim, S., Yu, H.R., Hwang, J., Kim, J.-I., and Kim, J.-S. (2015). Digenome-seq: genome-wide profiling of CRISPR-Cas9 off-target effects in human cells. Nat. Methods *12*, 237–243. https://doi.org/10.1038/nmeth.3284.

15. Cameron, P., Fuller, C.K., Donohoue, P.D., Jones, B.N., Thompson, M.S., Carter, M.M., Gradia, S., Vidal, B., Garner, E., Slorach, E.M., et al. (2017). Mapping the genomic landscape of CRISPR–Cas9 cleavage. Nat. Methods *14*, 600–606. https://doi.org/10.1038/nmeth.4284.

16. Lazzarotto, C.R., Malinin, N.L., Li, Y., Zhang, R., Yang, Y., Lee, G., Cowley, E., He, Y., Lan, X., Jividen, K., et al. (2020). CHANGE-seq reveals genetic and epigenetic effects on CRISPR–Cas9 genome-wide activity. Nat. Biotechnol. *38*, 1317–1327. https://doi.org/10.1038/s41587-020-0555-7.

17. Kim, D., and Kim, J.-S. (2018). DIG-seq: a genome-wide CRISPR off-target profiling method using chromatin DNA. Genome Res. *28*, 1894–1900. https://doi.org/10.1101/gr.236620.118.

18. European Medicines Agency (2020). Guideline on quality, non-clinical and clinical aspects of medicinal products containing genetically modified cells. https://www.ema.europa.eu/en/documents/scientific-guideline/guideline-quality-non-clinical-and-clinical-aspects-medicinal-products-containing-genetically-modified-cells-revision-1_en.pdf.

19. Food and Drug Administration (2024). Human gene therapy products incorporating human genome editing guidance for industry. https://www.fda.gov/media/156894/download.

20. Gee, P., Lung, M.S.Y., Okuzaki, Y., Sasakawa, N., Iguchi, T., Makita, Y., Hozumi, H., Miura, Y., Yang, L.F., Iwasaki, M., et al. (2020). Extracellular nanovesicles for packaging of CRISPR-Cas9 protein and sgRNA to induce therapeutic exon skipping. Nat. Commun. *11*, 1334. https://doi.org/10.1038/s41467-020-14957-y.

21. Kenjo, E., Hozumi, H., Makita, Y., Iwabuchi, K.A., Fujimoto, N., Matsumoto, S., Kimura, M., Amano, Y., Ifuku, M., Naoe, Y., et al. (2021). Low immunogenicity of LNP allows repeated administrations of CRISPR-Cas9 mRNA into skeletal muscle in mice. Nat. Commun. *12*, 7101. https://doi.org/10.1038/s41467-021-26714-w.

22. Pinello, L., Canver, M.C., Hoban, M.D., Orkin, S.H., Kohn, D.B., Bauer, D.E., and Yuan, G.-C. (2016). Analyzing CRISPR genome-editing experiments with CRISPResso. Nat. Biotechnol. *34*, 695–697. https://doi.org/10.1038/nbt.3583.

23. Maeder, M.L., Stefanidakis, M., Wilson, C.J., Baral, R., Barrera, L.A., Bounoutas, G.S., Bumcrot, D., Chao, H., Ciulla, D.M., DaSilva, J.A., et al. (2019). Development of a gene-editing approach to restore vision loss in Leber congenital amaurosis type 10. Nat. Med. *25*, 229–233. https://doi.org/10.1038/s41591-018-0327-9.

24. Abadi, S., Yan, W.X., Amar, D., and Mayrose, I. (2017). A machine learning approach for predicting CRISPR-Cas9 cleavage efficiencies and patterns underlying its mechanism of action. PLoS Comput. Biol. *13*, e1005807. https://doi.org/10.1371/journal.pcbi.1005807.

25. Yan, J., Xue, D., Chuai, G., Gao, Y., Zhang, G., and Liu, Q. (2020). Benchmarking and integrating genome-wide CRISPR off-target detection and prediction. Nucleic Acids Res. *48*, 11370–11379. https://doi.org/10.1093/nar/gkaa930.

26. Bae, S., Park, J., and Kim, J.-S. (2014). Cas-OFFinder: a fast and versatile algorithm that searches for potential off-target sites of Cas9 RNA-guided endonucleases. Bioinformatics *30*, 1473–1475. https://doi.org/10.1093/bioinformatics/btu048.

27. Montague, T.G., Cruz, J.M., Gagnon, J.A., Church, G.M., and Valen, E. (2014). CHOPCHOP: a CRISPR/Cas9 and TALEN web tool for genome editing. Nucleic Acids Res. *42*, W401–W407. https://doi.org/10.1093/nar/gku410.

28. O'Brien, A., and Bailey, T.L. (2014). GT-Scan: identifying unique genomic targets. Bioinformatics *30*, 2673–2675. https://doi.org/10.1093/bioinformatics/btu354.

29. Naito, Y., Hino, K., Bono, H., and Ui-Tei, K. (2015). CRISPRdirect: software for designing CRISPR/Cas guide RNA with reduced off-target sites. Bioinformatics *31*, 1120–1123. https://doi.org/10.1093/bioinformatics/btu743.

30. Pliatsika, V., and Rigoutsos, I. (2015). "Off-Spotter": very fast and exhaustive enumeration of genomic lookalikes for designing CRISPR/Cas guide RNAs. Biol. Direct *10*, 4. https://doi.org/10.1186/s13062-015-0035-z.

31. Park, J., Bae, S., and Kim, J.-S. (2015). Cas-Designer: a web-based tool for choice of CRISPR-Cas9 target sites. Bioinformatics *31*, 4014–4016. https://doi.org/10.1093/bioinformatics/btv537.

32. Perez, A.R., Pritykin, Y., Vidigal, J.A., Chhangawala, S., Zamparo, L., Leslie, C.S., and Ventura, A. (2017). GuideScan software for improved single and paired CRISPR guide RNA design. Nat. Biotechnol. *35*, 347–349. https://doi.org/10.1038/nbt.3804.

33. Zhu, L.J., Holmes, B.R., Aronin, N., and Brodsky, M.H. (2014). CRISPRseek: A bioconductor package to identify target-specific guide RNAs for CRISPR-Cas9 genome-editing systems. PLoS One *9*, e108424. https://doi.org/10.1371/journal.pone.0108424.

34. Cradick, T.J., Qiu, P., Lee, C.M., Fine, E.J., and Bao, G. (2014). COSMID: A web-based tool for identifying and validating CRISPR/Cas off-target sites. Mol. Ther. Nucleic Acids *3*, e214. https://doi.org/10.1038/mtna.2014.64.

35. Oliveros, J.C., Franch, M., Tabas-Madrid, D., San-León, D., Montoliu, L., Cubas, P., and Pazos, F. (2016). Breaking-Cas—interactive design of guide RNAs for CRISPR-Cas experiments for ENSEMBL genomes. Nucleic Acids Res. *44*, W267–W271. https://doi.org/10.1093/nar/gkw407.

36. Concordet, J.-P., and Haeussler, M. (2018). CRISPOR: intuitive guide selection for CRISPR/Cas9 genome editing experiments and screens. Nucleic Acids Res. *46*, W242–W245. https://doi.org/10.1093/nar/gky354.

37. Zhang, D., Hurst, T., Duan, D., and Chen, S.-J. (2019). Unified energetics analysis unravels SpCas9 cleavage activity for optimal gRNA design. Proc. Natl. Acad. Sci. USA *116*, 8693–8698. https://doi.org/10.1073/pnas.1820523116.

38. Alkan, F., Wenzel, A., Anthon, C., Havgaard, J.H., and Gorodkin, J. (2018). CRISPR-Cas9 off-targeting assessment with nucleic acid duplex energy parameters. Genome Biol. *19*, 177. https://doi.org/10.1186/s13059-018-1534-x.

39. Benjamin, D., Sato, T., Cibulskis, K., Getz, G., Stewart, C., and Lichtenstein, L. (2019). Calling somatic SNVs and indels with Mutect2. Preprint at Bioinformatics. https://doi.org/10.1101/861054.

40. Chen, X., Schulz-Trieglaff, O., Shaw, R., Barnes, B., Schlesinger, F., Källberg, M., Cox, A.J., Kruglyak, S., and Saunders, C.T. (2016). Manta: rapid detection of structural variants and indels for germline and cancer sequencing applications. Bioinformatics *32*, 1220–1222. https://doi.org/10.1093/bioinformatics/btv710.

41. Hashimoto, N., Kiyono, T., Wada, M.R., Shimizu, S., Yasumoto, S., and Inagawa, M. (2006). Immortalization of human myogenic progenitor cell clone retaining multipotentiality. Biochem. Biophys. Res. Commun. *348*, 1383–1388. https://doi.org/10.1016/j.bbrc.2006.08.006.

42. Tate, J.G., Bamford, S., Jubb, H.C., Sondka, Z., Beare, D.M., Bindal, N., Boutselakis, H., Cole, C.G., Creatore, C., Dawson, E., et al. (2019). COSMIC: the catalogue of somatic mutations in cancer. Nucleic Acids Res. *47*, D941–D947. https://doi.org/10.1093/nar/gky1015.

43. Pharmaceuticals and Medical Devices Agency (2013). Current perspective on evaluation of tumorigenicity of cellular and tissue-based products derived from induced pluripotent stem Cells (iPSCs) and iPSCs as their starting materials. http://www.pmda.go.jp/files/000152599.pdf.

44. Eguchi, M., Eguchi-Ishimae, M., and Greaves, M. (2004). The small oligomerization domain of gephyrin converts MLL to an oncogene. Blood *103*, 3876–3882. https://doi.org/10.1182/blood-2003-11-3817.

45. Kraft, K., Geuer, S., Will, A.J., Chan, W.L., Paliou, C., Borschiwer, M., Harabula, I., Wittler, L., Franke, M., Ibrahim, D.M., et al. (2015). Deletions, inversions, duplications: engineering of structural variants using CRISPR/Cas in mice. Cell Rep. *10*, 833–839. https://doi.org/10.1016/j.celrep.2015.01.016.

46. Aparicio-Prat, E., Arnan, C., Sala, I., Bosch, N., Guigó, R., and Johnson, R. (2015). DECKO: Single-oligo, dual-CRISPR deletion of genomic elements including long non-coding RNAs. BMC Genom. *16*, 846. https://doi.org/10.1186/s12864-015-2086-z.

47. Li, J., Shou, J., Guo, Y., Tang, Y., Wu, Y., Jia, Z., Zhai, Y., Chen, Z., Xu, Q., and Wu, Q. (2015). Efficient inversions and duplications of mammalian regulatory DNA elements and gene clusters by CRISPR/Cas9. J. Mol. Cell Biol. *7*, 284–298. https://doi.org/10.1093/jmcb/mjv016.

48. Binda, C.S., Klaver, B., Berkhout, B., and Das, A.T. (2020). CRISPR-Cas9 dual-gRNA attack causes mutation, excision and inversion of the HIV-1 proviral DNA. Viruses *12*, 330. https://doi.org/10.3390/v12030330.

49. Ono, R., Ishii, M., Fujihara, Y., Kitazawa, M., Usami, T., Kaneko-Ishino, T., Kanno, J., Ikawa, M., and Ishino, F. (2015). Double strand break repair by capture of retrotransposon sequences and reverse-transcribed spliced mRNA sequences in mouse zygotes. Sci. Rep. *5*, 12281. https://doi.org/10.1038/srep12281.

50. Tao, J., Wang, Q., Mendez-Dorantes, C., Burns, K.H., and Chiarle, R. (2022). Frequency and mechanisms of LINE-1 retrotransposon insertions at CRISPR/Cas9 sites. Nat. Commun. *13*, 3685. https://doi.org/10.1038/s41467-022-31322-3.

51. Bao, X.R., Pan, Y., Lee, C.M., Davis, T.H., and Bao, G. (2021). Tools for experimental and computational analyses of off-target editing by programmable nucleases. Nat. Protoc. *16*, 10–26. https://doi.org/10.1038/s41596-020-00431-y.

52. Suzuki, K., Yu, C., Qu, J., Li, M., Yao, X., Yuan, T., Goebl, A., Tang, S., Ren, R., Aizawa, E., et al. (2014). Targeted gene correction minimally impacts whole-genome mutational load in human-disease-specific induced pluripotent stem cell clones. Cell Stem Cell *15*, 31–36. https://doi.org/10.1016/j.stem.2014.06.016.

53. Veres, A., Gosis, B.S., Ding, Q., Collins, R., Ragavendran, A., Brand, H., Erdin, S., Cowan, C.A., Talkowski, M.E., and Musunuru, K. (2014). Low Incidence of off-target mutations in individual CRISPR-Cas9 and TALEN targeted human stem cell clones detected by whole-genome sequencing. Cell Stem Cell *15*, 27–30. https://doi.org/10.1016/j.stem.2014.04.020.

54. Smith, C., Gore, A., Yan, W., Abalde-Atristain, L., Li, Z., He, C., Wang, Y., Brodsky, R.A., Zhang, K., Cheng, L., and Ye, Z. (2014). Whole-genome sequencing analysis reveals high specificity of CRISPR/Cas9 and TALEN-based genome editing in human iPSCs. Cell Stem Cell *15*, 12–13. https://doi.org/10.1016/j.stem.2014.06.011.

55. Yang, L., Grishin, D., Wang, G., Aach, J., Zhang, C.-Z., Chari, R., Homsy, J., Cai, X., Zhao, Y., Fan, J.-B., et al. (2014). Targeted and genome-wide sequencing reveal single nucleotide variations impacting specificity of Cas9 in human stem cells. Nat. Commun. *5*, 5507. https://doi.org/10.1038/ncomms6507.

56. Smith, R.H., Chen, Y.-C., Seifuddin, F., Hupalo, D., Alba, C., Reger, R., Tian, X., Araki, D., Dalgard, C.L., Childs, R.W., et al. (2020). Genome-wide analysis of off-target CRISPR/Cas9 activity in single-cell-derived human hematopoietic stem and progenitor cell clones. Genes *11*, 1501. https://doi.org/10.3390/genes11121501.

57. Kita, Y., Okuzaki, Y., Naoe, Y., Lee, J., Bang, U., Okawa, N., Ichiki, A., Jonouchi, T., Sakurai, H., Kojima, Y., and Hotta, A. (2023). Dual CRISPR-Cas3 system for

inducing multi-exon skipping in DMD patient-derived iPSCs. Stem Cell Rep. *18*, 1753–1765. https://doi.org/10.1016/j.stemcr.2023.07.007.

58. Kosicki, M., Tomberg, K., and Bradley, A. (2018). Repair of double-strand breaks induced by CRISPR–Cas9 leads to large deletions and complex rearrangements. Nat. Biotechnol. *36*, 765–771. https://doi.org/10.1038/nbt.4192.

59. Tan, E., Li, Y., Del Castillo Velasco-Herrera, M., Yusa, K., and Bradley, A. (2015). Off-target assessment of CRISPR-Cas9 guiding RNAs in human iPS and mouse ES cells. Genesis *53*, 225–236. https://doi.org/10.1002/dvg.22835.

60. Kuwada, N., Kimura, F., Matsumura, T., Yamashita, T., Nakamura, Y., Wakimoto, N., Ikeda, T., Sato, K., and Motoyoshi, K. (2001). t(11;14)(q23;q24) generates an MLL-human gephyrin fusion gene along with a de facto truncated MLL in acute monoblastic leukemia. Cancer Res. *61*, 2665–2669.

61. Eguchi, M., Eguchi-Ishimae, M., Seto, M., Morishita, K., Suzuki, K., Ueda, R., Ueda, K., Kamada, N., and Greaves, M. (2001). *GPHN*, a novel partner gene fused to *MLL* in a leukemia With t(11;14)(q23;q24). Genes Chromosomes Cancer *32*, 212–221. https://doi.org/10.1002/gcc.1185.

62. Yen, A., Zappala, Z., Fine, R.S., Majarian, T.D., Sripakdeevong, P., and Altshuler, D. (2024). Specificity of CRISPR-Cas9 editing in exagamglogene autotemcel. N. Engl. J. Med. *390*, 1723–1725. https://doi.org/10.1056/NEJMc2313119.

63. Gillmore, J.D., Gane, E., Taubel, J., Kao, J., Fontana, M., Maitland, M.L., Seitzer, J., O'Connell, D., Walsh, K.R., Wood, K., et al. (2021). CRISPR-Cas9 in vivo gene editing for transthyretin amyloidosis. N. Engl. J. Med. *385*, 493–502. https://doi.org/10.1056/NEJMoa2107454.

64. Frangoul, H., Altshuler, D., Cappellini, M.D., Chen, Y.-S., Domm, J., Eustace, B.K., Foell, J., De La Fuente, J., Grupp, S., Handgretinger, R., et al. (2021). CRISPR-Cas9 gene editing for sickle cell disease and β-thalassemia. N. Engl. J. Med. *384*, 252–260. https://doi.org/10.1056/NEJMoa2031054.

OMTN, Volume *37*

Supplemental information

Comprehensive assessment of on- and off-target mutagenesis via lipid nanoparticle delivery of CRISPR-Cas9 genome editing

Youichi Naoe, Naoko Fujimoto, Yukimasa Makita, Dongyang Li, Naoto Inukai, and Akitsu Hotta

Figure S1

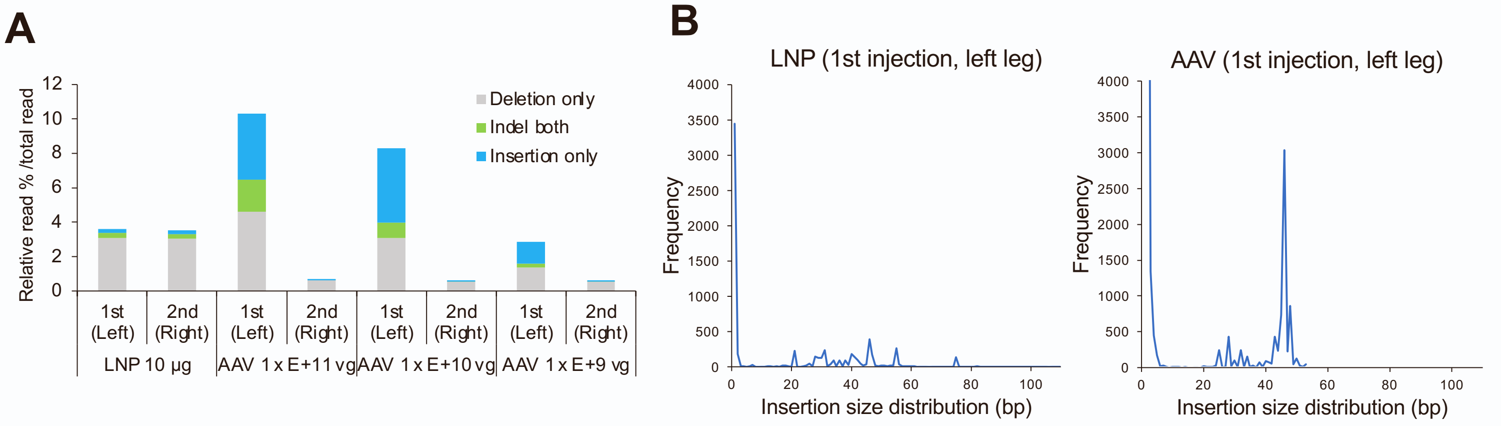

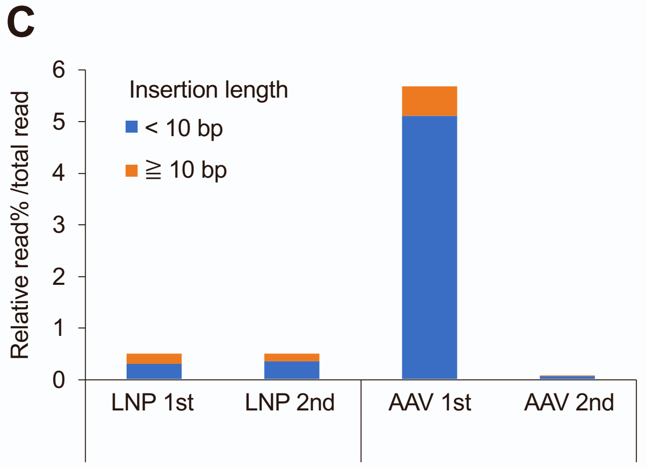

D

	LNP		AAV
	1st	2nd	1st
Total insertion reads (≧ 10 bp)	2,942	2,315	8,726
DMD exon 45 reads	1,579	1,332	5,091
Inverted *DMD* exon 45 reads	1,576	1,324	5,076
Percentage of inverted reads at *DMD* exon 45	99.8%	99.4%	99.7%

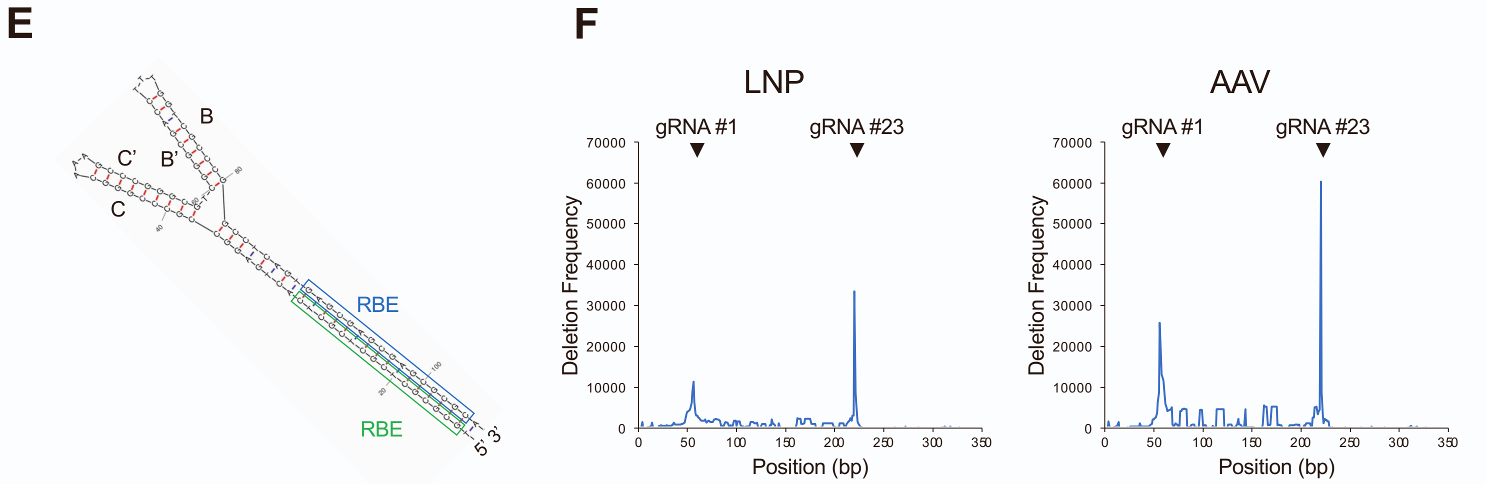

Figure S1: Amplicon-seq analysis of on-target site in CAG-Luc2-hDMD Ex45 knock-in reporter mice.

A. Comparison of indel event proportions between LNP and AAV at various doses.

B. Size distribution of the insertion sequences. CAG-Luc2-hDMD Ex45 knock-in mice were administered with LNP-CRISPR (10 µg) or AAV9-CRISPR (1 × 10^{11} v.g.). The first injection was on the left gastrocnemius (GC) muscle, and the second injection was on the right GC muscle. After genome DNA extraction, the on-target region of the DMD exon 45 was PCR amplified and analyzed by amplicon-seq. Among the detected indel patterns, only the insertion sequences were extracted, and their sizes are plotted.

C. Among the insertion sequences, the ratio of sequences longer than 10 bp or equal to 10 bp is indicated in orange, and shorter than 10 bp in blue.

D. Out of the detected human *DMD* exon 45 sequence from the insertion sequences, the percentage of inversion is indicated.

E. Schematic of DNA secondary structure of the ITR (inverted terminal repeat) region of the AAV vector backbone sequence. Rep binding elements (RBEs) and palindromic arms (B-B' and C-C') are indicated.

F. Deletion frequency at each position relative to the two gRNA target sites. Left: LNP-CRISPR-injected mice. Right: AAV-CRISPR-injected mice.

Figure S2

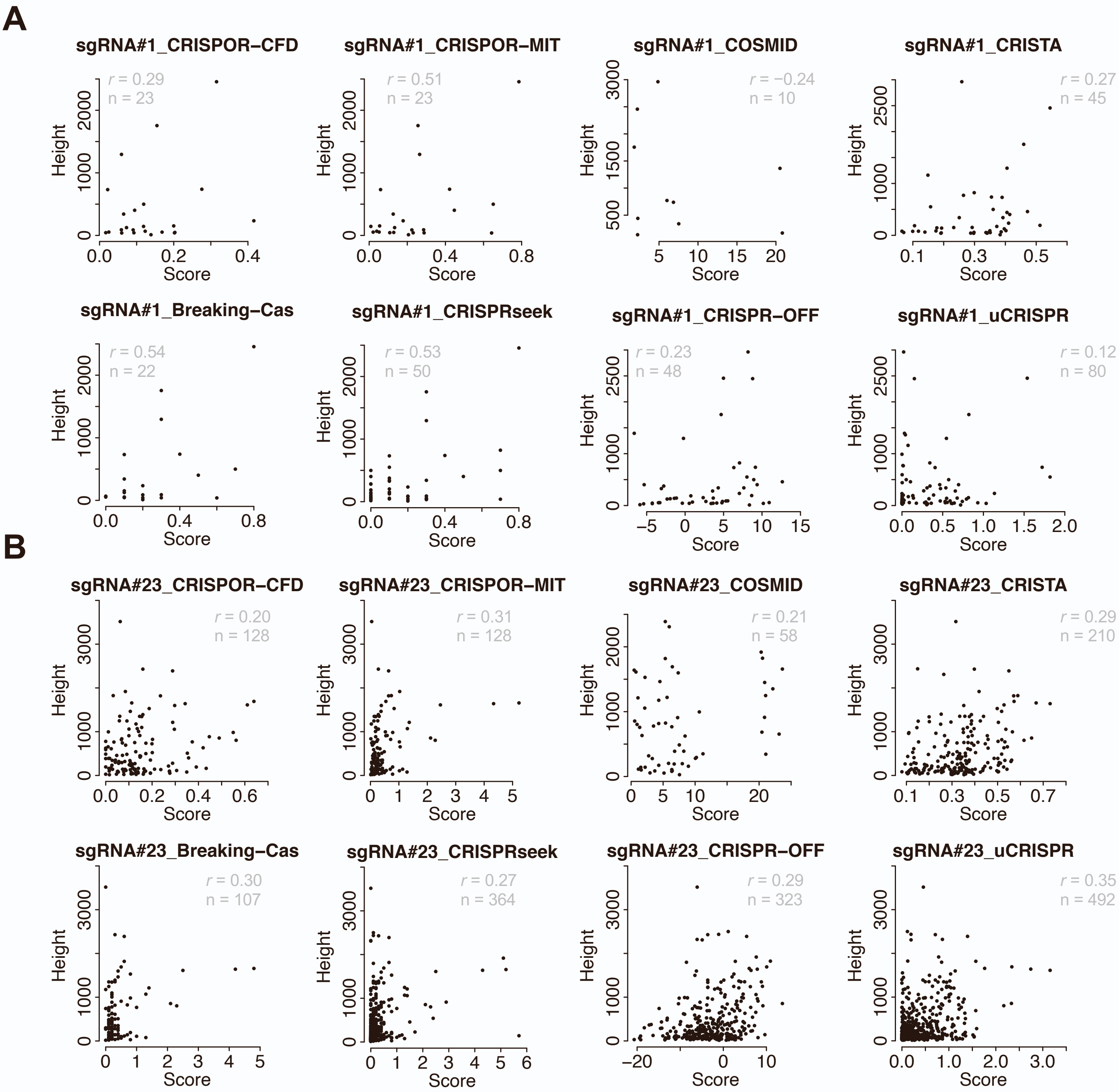

Figure S2: Performance evaluation of *in silico* prediction tools for potential off-target binding/cleavage sites.
A. The sequence of the gRNA #1 was provided to each *in silico* prediction tool, and potential off-target sites were obtained. After extracting the overlapping site with our CIRCLE-seq experimental results, the output specificity scores from the tools were plotted against the CIRCLE-seq peak heights, as an indicator of potential DNA cleavage activity *in vitro*. The number of overlapping sites is shown as "n", and the correlation coefficient is shown as "*r*". For CRISPOR, both the CFD Specificity score and the MIT Guide Specificity score were utilized for plotting.
B. Scattered plots of the tool specificity scores against the CIRCLE-seq peak heights by using the gRNA #23

Figure S3

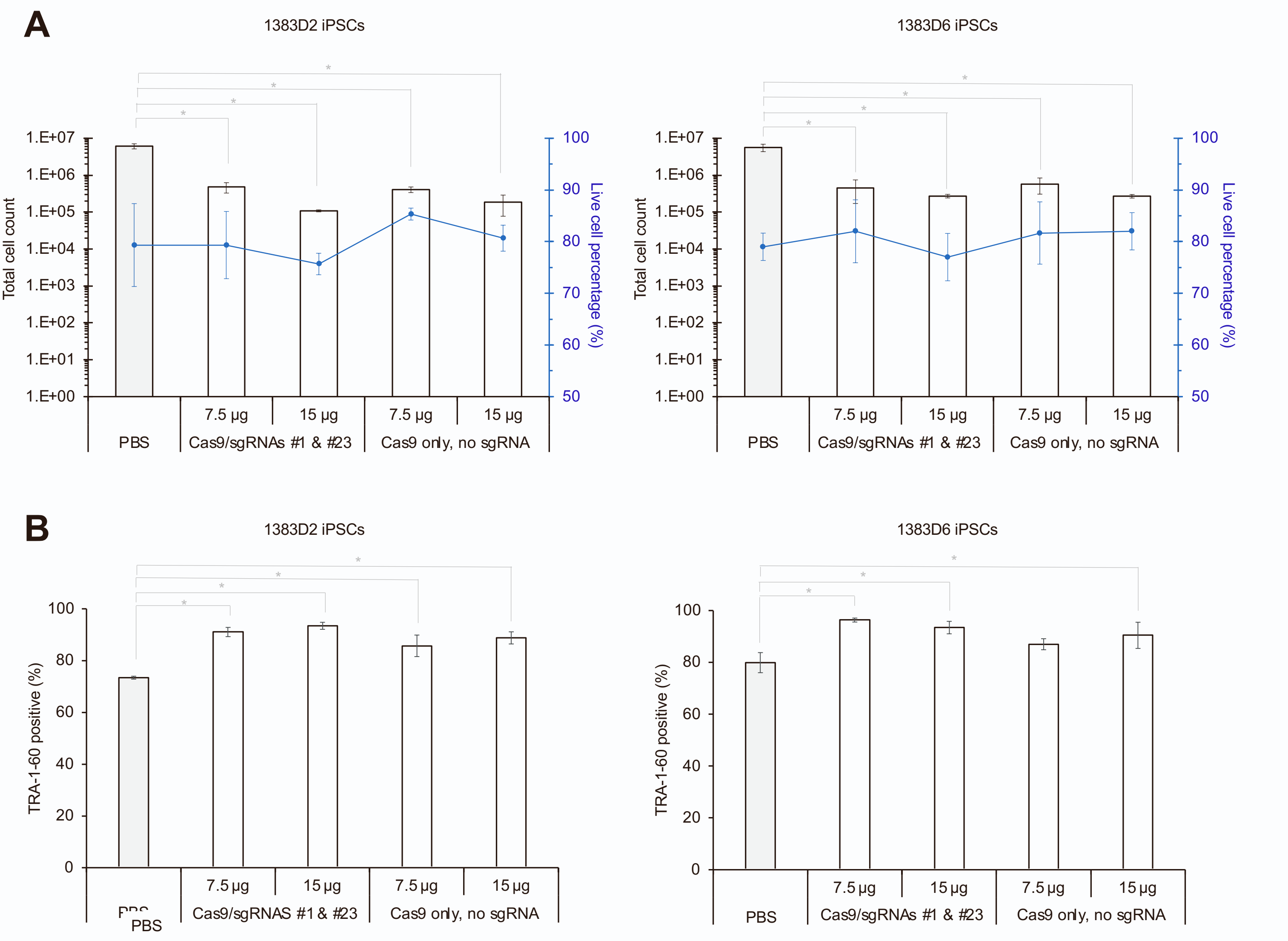

Figure S3: Effects of LNP on cell viability and pluripotency in iPSCs.

A. Effects of LNP on iPS cell viability. The left y-axis (black) shows total cell count and right y-axis (blue) shows live cell percentage, both presented as mean ± S.D. (n = 3 wells), and the x-axis represents the experimental conditions. Total RNA amount at two levels (7.5 µg and 15 µg) were used to treat iPSC in the presence or absence of gRNA, and the results were compared with the PBS group. The left panel shows iPSC line 1383D2, and the right panel shows 1383D6. Statistical significance was determined by one-way ANOVA followed by Dunnett's multiple comparison test versus PBS. * $p < 0.05$. No significant differences were observed for live cell percentage (right y-axis).

B. Effects of LNP treatment on pluripotency in iPSC lines 1383D2 and 1383D6. The y-axis indicates TRA-1-60 positive cell percentage of iPSCs assessed by flow cytometry shown as mean ± S.D. (n = 3 wells). Note that PBS treated iPS cells were overconfluent and therefore exhibited slightly lower TRA-1-60 positiveness. Statistical significance was determined by one-way ANOVA followed by Dunnett's multiple comparison test versus PBS. * $p < 0.05$.

Figure S4

A

iPSC line	Treated gRNA	Total seq. reads	Mapped reads	Mean depth
1383D2	-	1,550,594,738	96.5%	79.25
	#1	1,389,839,194	96.6%	71.03
	#23	1,477,430,064	96.7%	75.51
	#1 + #23	1,555,112,436	96.6%	79.48
1383D6	-	1,269,887,554	96.5%	64.90
	#1	1,284,051,876	96.5%	65.63
	#23	1,591,064,548	96.4%	81.32
	#1 + #23	1,205,774,840	96.5%	61.63

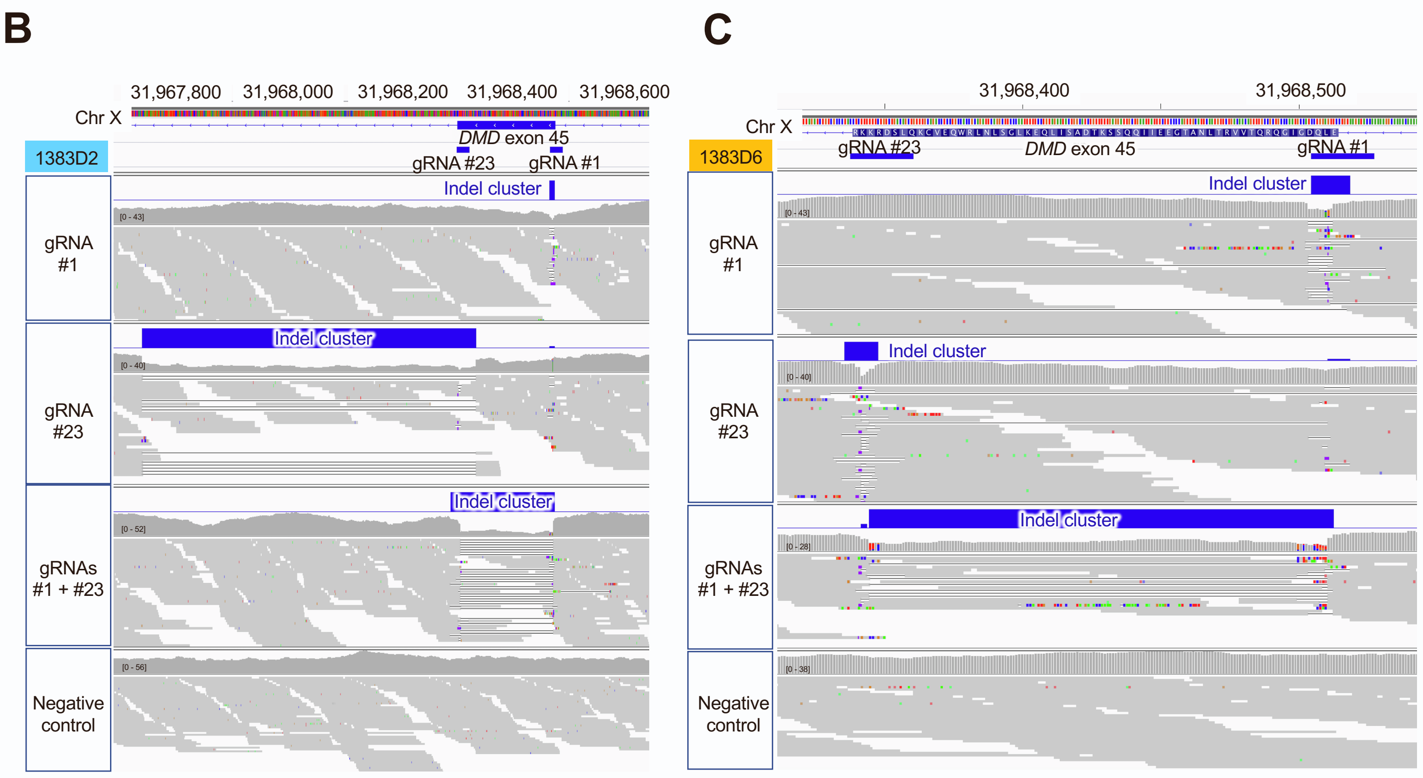

Figure S4: Summary of whole genome sequencing analysis and on-target confirmation.
A. Summary statistics of the sequencing and mapping results for human iPSCs treated with LNP-CRISPR.
B. Validation of the on-target genome editing from the WGS analysis. iPSC 1383D2 line was treated with LNP-CRISPR delivering gRNA #1 only, gRNA #23 only, or a combination of gRNAs #1 and #23. Mapped sequence reads at the *DMD* gene exon 45 region are shown in light gray bars at the bottom, coverage depths are shown in dark gray peaks at the middle, and the "indel clusters" detected *de novo* from the negative control (in Figure 3F) are shown in a blue bar graph at the top. For the negative control, only mapped sequence reads are shown.
C. Similar to above, the iPSC 1383D6 line was treated with LNP-CRISPR and analyzed by WGS.

Figure S5

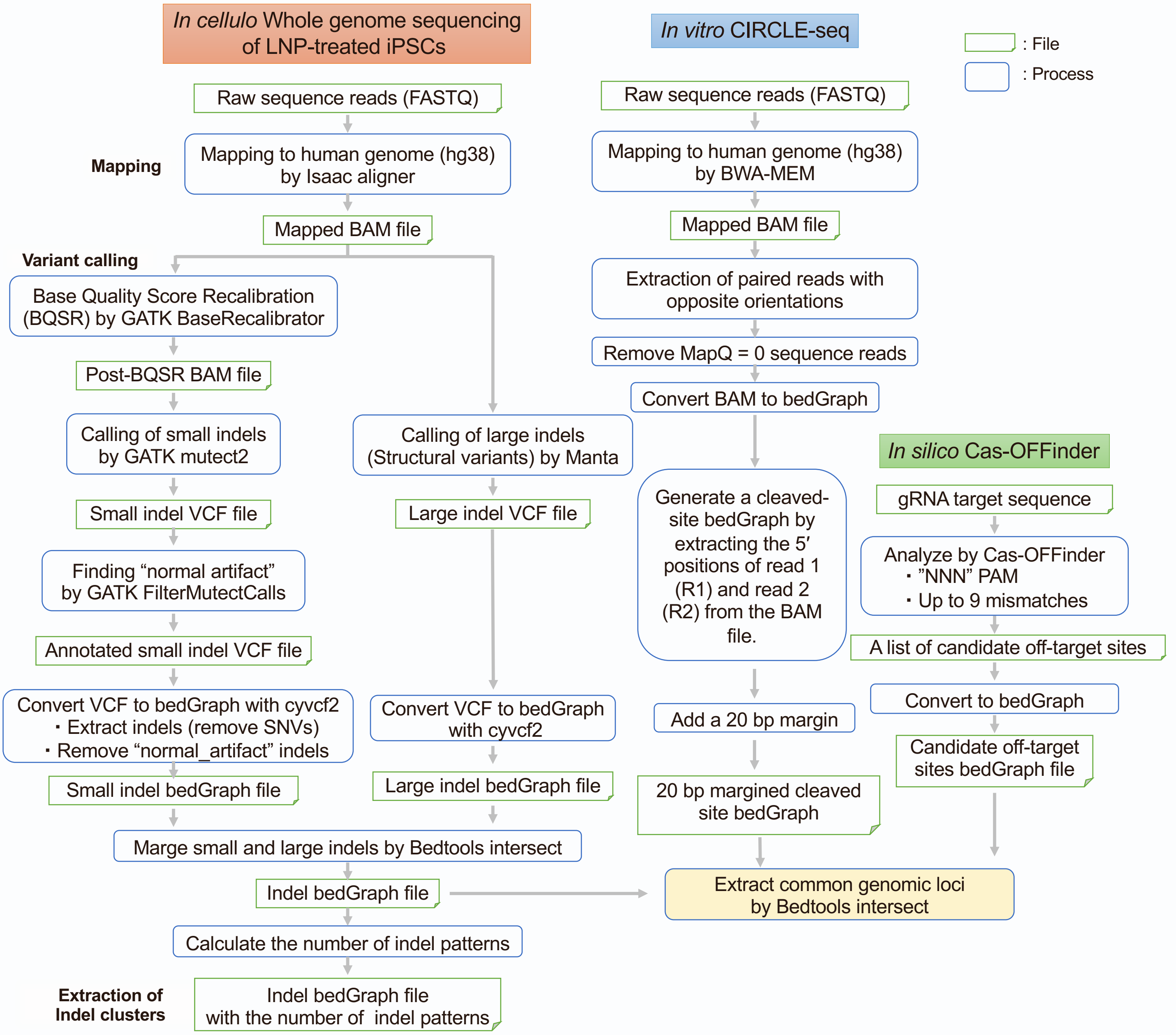

Figure S5: The analytical flowchart of the data integration of WGS, CIRCLE-seq, and Cas-OFFinder data.
File format is indicated by a light green rectangle with a folded corner, and process is indicated by a light blue rounded rectangle.

Figure S6

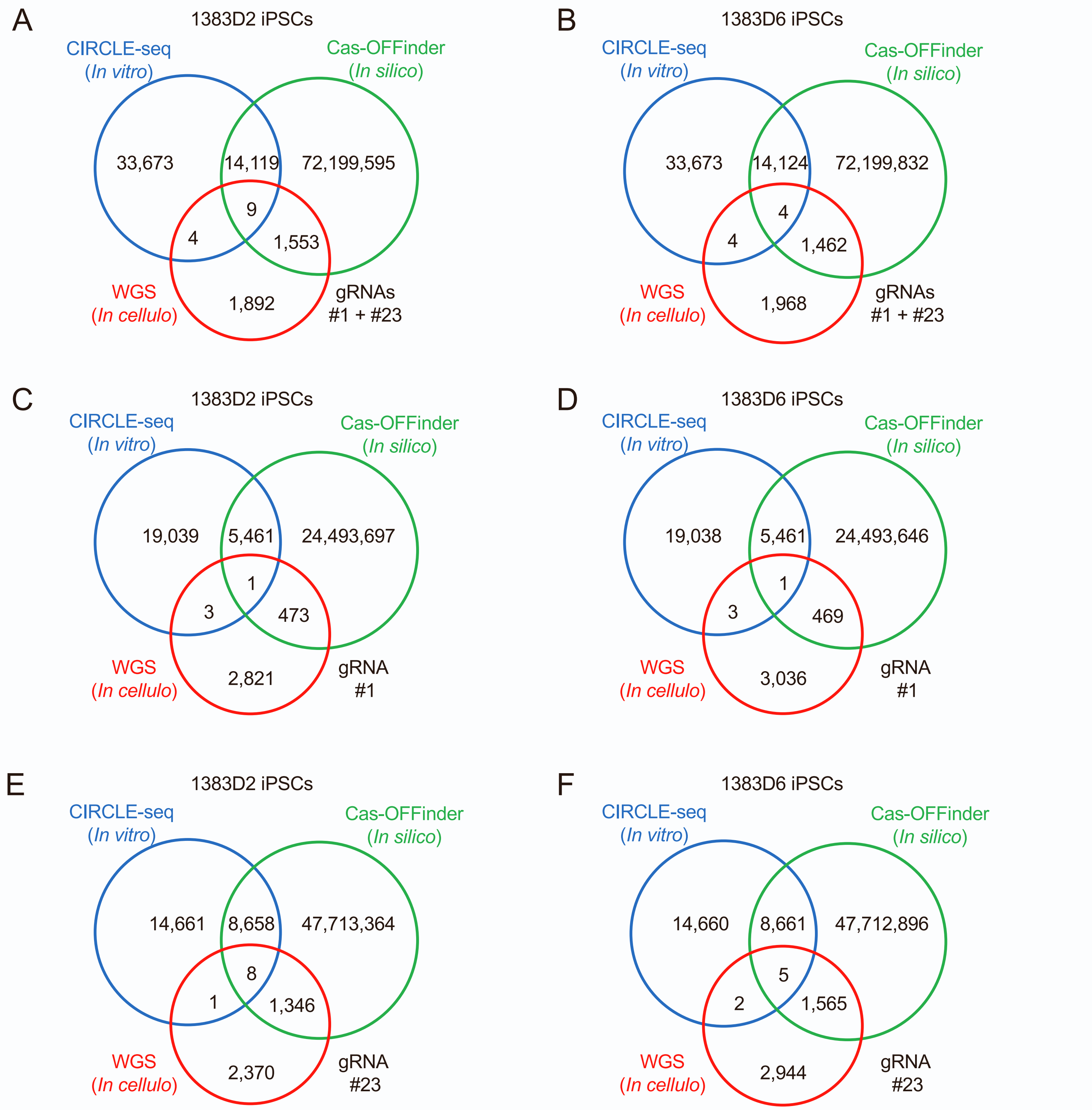

Figure S6: Venn diagram of the combined off-target sites of WGS, CIRCLE-seq, and Cas-OFFinder analyses. Related to Figure 4B, the number of overlapping sites is indicated in the Venn diagram. Left side (A, C, E): data from 1383D2 iPSCs, right side (B, D, F): data from 1383D6 iPSCs. Top row (A, B): WGS data with gRNA #1 and #23 treatment, middle row (C, D): WGS data with gRNA #1 only, bottom row (E, F): WGS data with gRNA #23 only. Notably, all the on-target sites were detected as common from the three analysis methods.

Figure S7

A

Primer set ID (gRNA position)	Forward 5'-3'	Reverse 5'-3'	Annealing temp. (° C)	PCR product size (bp)
chrX:31968338-31968527 (On-target)	AGTGCCTTTCACCCTGCTTA	GGGCATGTCAGTTTCATAGGGA	60	432
chr1:17729009-17729031	CGAGTGGGAGGAGATGAAATAGG	CTTGAACAAGTCAGGAGACCTCA	60	447
chr1:84361946-84361968	CTGAAGGGAAGAGGAGCTTATGT	AAGACTCCAGGGGAGATTCTGT	60	406
chr3:67017674-67017696	CTCACTTGGTGTTTGTGTAGCTG	AGTGCCCAGTAAAGTGTGGATAG	60	489
chr5:113937846-113937868	GACAGAGCACTTATGTTTCAGGC	TGTTTCCCACCTGTGTTCCATAT	60	492
chr6:137734937-137734959	ACAGGGAAAAGCCAAGCCTTAC	CCCATCACAAGGAGGAAGTAGG	60	475
chr7:140404449-140404471	GGTAAGCGACAGGTGAACCA	CCCTGCCTTGTGACATCCTT	60	484
chr9:11687303-11687325	GGATTCCCTCCCCAGACAAAC	CCATGGTACTGAGTGGGTGAA	60	432
chr14:67492431-67492453	TGTGTTACTGTAAAGAGCTGCCT	TAGAAATCTCTAGGTGGCTTGGC	63	408
chr18:58827057-58827079	TTTCCAGGGAAAGGAAACCCAG	AGAACTGTACACTCACCGCG	63	460
chr20:58378315-58378337	ATGCCAATAAGGTGCTGGAC	TTTGCATTTCCTCAGCATCTACT	60	400
chrX:96288780-96288802	AAAGGACATCTCACCGTTGTCT	TCTGAGTGGCAACTAAATGGGTC	60	400

B

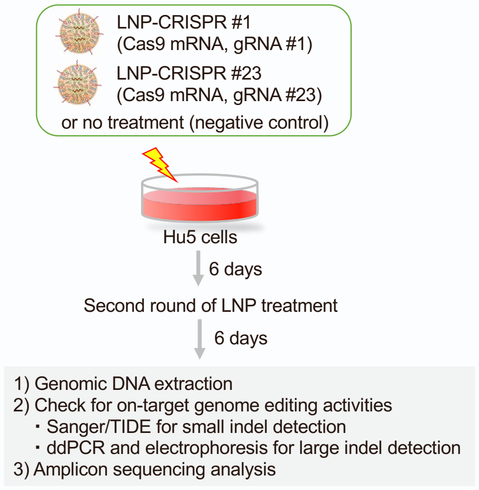

C

Small indel % by TIDE assay	Hu5	
gRNA	#1	#23
Indel (%)	63.9	67.7

D

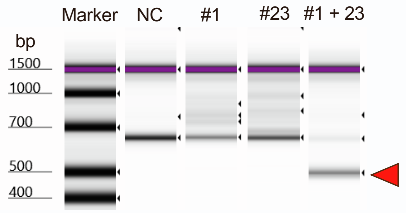

Figure S7: Primer list for amplicon sequencing and and preparation of human myogenic Hu5 cells.
A. Primer pairs used to amplify the candidate off-target loci, related to Figure 4D. The on-target site highlighted in light beige was incorporated as positive control. "Annealing temp. (℃)" indicates the PCR annealing temperature used for amplicon sequencing library preparation to minimize non-specific amplifications.
B. Experimental workflow for genome editing in Hu5 cells using LNP-mediated CRISPR-Cas9 delivery.
C. Small indel frequencies at the on-target site determined by TIDE analysis of Sanger sequencing data in Hu5 cells following LNP-CRISPR treatment with gRNA #1 or gRNA #23.
D. Representative TapeStation electrophoresis image of PCR-amplified on-target regions in Hu5 cells. NC indicates untreated negative control. The red arrow indicates the deletion fragment generated between the two CRISPR target sites.

Figure S8

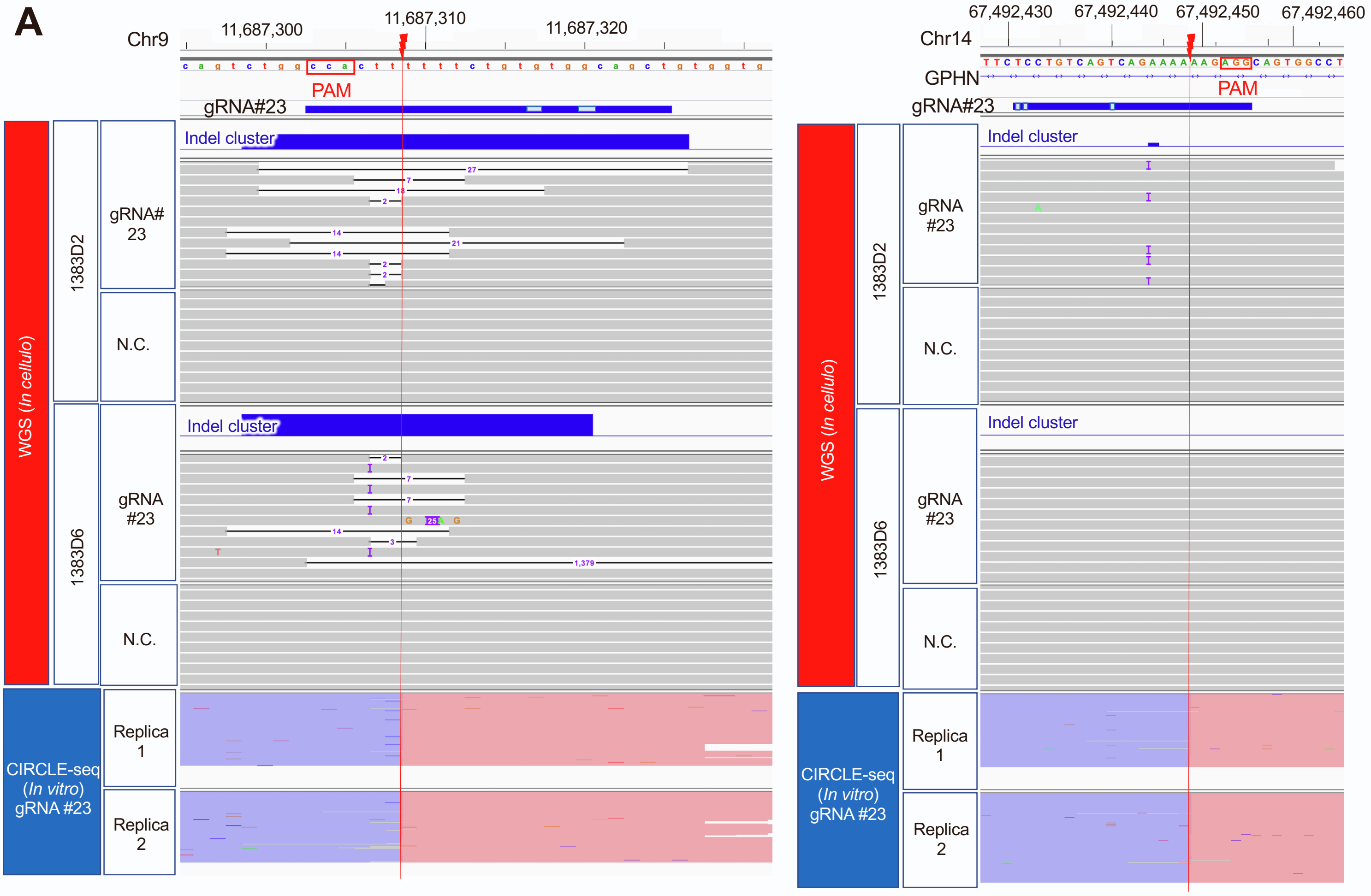

B

gRNA-independent off-target		WGS_off-target_site	Allele Frac	Indel pattern	WGS Genome_edited Mean_MapQ	WGS Genome_edited No._Reads	WGS NC Mean_MapQ	WGS NC No._Reads	CIRCLE-seq Peak height	CIRCLE-seq Used gRNA	Repeat element	Problematic region	Mappability
1383D2 iPSC	gRNA #1	chr4_120645206_120645206	0.061	1	53.87	94	54.37	99	0, 18	#1	LINE	Yes	0
		chr8_51696703_51696703	0.046	1	44.92	148	45.15	170	0, 30	#1	-	Yes	0.71
		chr16_34581526_34581528	0.003	1	28.86	10602	29.39	12094	65, 109	#1	-	Yes	0
	#23	chr4_49637782_49637784	0.153	1	22.36	1168	23.59	1310	2, 23	#23	Satellite	Yes	0.08
	#1 + #23	chr4_49637782_49637784	0.086	1	22.17	1191	23.59	1310	2, 23	#23	Satellite	Yes	0.08
		chr9_63664629_63664629	0.056	1	38.69	140	40.66	109	0, 11	#1	Simple repeat	Yes	0.13
		chr22_11813420_11813420	0.014	1	12.7	5018	12.08	5057	11, 0	#23	Simple repeat	Yes	0
		chr22_26210002_26210002	0.160	1	55.22	118	57.03	128	0, 10	#23	Low complexity	Yes	0.67
1383D6 iPSC	#1	chr9_63664618_63664627	0.075	1	35.94	118	40.29	105	0, 11	#1	Simple repeat	Yes	0.0375
		chr16_46387236_46387257	0.003	1	21.32	27246	21.59	26199	0, 22	#1	-	Yes	0
		chr21_9248177_9248178	0.016	1	52.3	1167	51.68	1137	0, 13	#1	-	Yes	0.72916
	#23	chr1_153505473_153505473	0.036	1	60	167	60	100	21, 0	#23	SINE	No	0.29
		chr14_52845949_52846136	0.021	1	56.58	205	57.37	137	0, 19	#23	SINE	Partially Yes	0.154
	#1 + #23	chr2_117780613_117780614	0.079	1	57.77	95	58.1	103	0, 34	#23	LINE	Yes	0
		chr7_61048180_61048180	0.023	1	57.86	355	56.55	347	0, 28	#1	-	Yes	0.29
		chr10_124227297_124227348	0.033	1	52.76	259	54.25	295	28, 0	#23	-	Yes	0.664
		chrY_20278947_20278947	0.045	1	54.88	159	55.94	174	0, 66	#1	LTR	Yes	0

On-target		WGS_off-target_site	Allele Frac	Indel pattern	Genome_edited Mean MapQ	Genome_edited No. Reads	NC Mean MapQ	NC No. Reads	Peak height	Used gRNA	Repeat element	Problematic region	Mappability
1383D2 iPSC	#1	chrX_31968504_31968512	1.810	3	60	45	60	62	1562, 5245	#1	-	No	1
	#23	chrX_31967773_31968371	1.688	5	59.31	87	60	221	2023, 3129	#23	-	Partially Yes	1
	#1 + #23	chrX_31968326_31968512	1.522	5	58.04	92	60	118	2023, 3129	#23	-	No	1
		chrX_31968326_31968512	1.522	5	58.04	92	60	118	1562, 5245	#1	-	No	1
1383D6 iPSC	#1	chrX_31968504_31968518	1.599	4	60	68	60	62	1562, 5245	#1	-	No	1
	#23	chrX_31968335_31968347	1.064	7	59.96	61	60	60	2023, 3129	#23	-	No	1
	#1 + #23	chrX_31968341_31968343	0.368	2	58.28	35	60	58	2023, 3129	#23	-	No	1
		chrX_31968344_31968512	1.656	4	58.88	54	60	96	2023, 3129	#23	-	No	1
		chrX_31968344_31968512	1.656	4	58.88	54	60	96	1562, 5245	#1	-	No	1

Figure S8: Detailed analysis of the off-target sites detected.

A. Two representative off-target sites detected from the comprehensive analysis. Left: chr9:11687303-11687325 in iPSCs 1383D2 and 1383D6 treated with gRNA #23. Right: chr14:67492431-67492453 in iPSC 1383D2 treated with gRNA #23. The mismatch positions on the gRNA sequence are shown in light blue. The red lightning mark indicates a Cas9 cleavage site. The CIRCLE-seq reads shown in blue and red represent the minus and plus strands, respectively, and the junction indicates the DNA cleavage site.

B. Putative gRNA-independent off-target sites that were detected by experimental WGS and CIRCLE-seq, but not by *in silico* analysis (upper). Repeat element (RepeatMasker), Problematic regions, and Mappability are annotated based on the UCSC Genome browser. For the Mean MapQ and Mappability, the deeper color indicates a higher value. A table similar to the one above for the on-target sites (lower).
